# The Impact of Exosomes/Microvesicles Derived from Myeloid Dendritic Cells Cultured in the Presence of Calcitriol and Tacalcitol on Acute B-Cell Precursor Cell Lines with *MLL* Fusion Gene

**DOI:** 10.3390/jcm11082224

**Published:** 2022-04-15

**Authors:** Eliza Turlej, Tomasz Marek Goszczyński, Marek Drab, Beata Orzechowska, Magdalena Maciejewska, Joanna Banach, Joanna Wietrzyk

**Affiliations:** 1 Department of Experimental Biology, Wroclaw University of Environmental and Life Science, 50-375 Wroclaw, Poland; 2 Laboratory of Biomedical Chemistry, Hirszfeld Institute of Immunology and Experimental Therapy, Polish Academy of Sciences, 53-114 Wroclaw, Poland; tomasz.goszczynski@hirszfeld.pl; 3 Laboratory of Interactions of Biological Nanostructures, Department of Immunology of Infectious Diseases, Hirszfeld Institute of Immunology and Experimental Therapy, Polish Academy of Sciences, 53-114 Wroclaw, Poland; marek.drab@hirszfeld.pl; 4 Laboratory of Virology, Department of Immunology of Infectious Diseases, Hirszfeld Institute of Immunology and Experimental Therapy, Polish Academy of Sciences, 53-114 Wroclaw, Poland; beata.orzechowska@hirszfeld.pl; 5 Department of Experimental Oncology, Hirszfeld Institute of Immunology and Experimental Therapy, Polish Academy of Sciences, 53-114 Wroclaw, Poland; magdalena.maciejewska@hirszfeld.pl (M.M.); joanna.banach@hirszfeld.pl (J.B.); joanna.wietrzyk@hirszfeld.pl (J.W.)

**Keywords:** immature myeloid-derived dendritic cells, microvesicles, exosomes, acute lymphoblastic B leukemia, B cells, *MLL* translocation, calcitriol, tacalcitol

## Abstract

Vitamin D analogs (VDAs) may directly inhibit the growth of normal and malignant (derived from acute lymphoblastic leukemia (ALL)) B cells, as both types of cells express vitamin D receptor (VDR). We performed anti-proliferative, morphology tests and phenotyping to evaluate the sensitivity of monocytes and iDCs (immature myeloid-derived dendritic cells) on calcitriol and tacalcitol treatment, phenotyping, morphology, and size distribution measurement to determine the characteristics of microvesicles (MVs) and exosomes (EXs) derived from them and, finally, phenotyping and Elisa test to determine the effects of VDAs on modulation of the phenotype of B cells through extracellular vesicles (EVs) released by iDCs. Our results confirmed that both SC cells and iDCs were sensitive to the VDAs and showed altered surface expression of markers associated with monocyte differentiation, which was resulting in the phenotypic changes in EVs derived from them. We also showed that obtained EVs could change the morphology and phenotype of ALL-B-derived precursor cells in a different way, depending on their origin. The differential effect of VDAs on ALL-B cells, which was associated with increased or decreased expression of CD27, CD24, CD38, and CD23 expression, was observed. Hence, further studies to explain the modulation in the composition of EVs by VDAs are required.

## 1. Introduction

Among the antigen-presenting cells (APCs), dendritic cells (DCs) represent a heterogeneous cell population in terms of their origin, localization, phenotype, and function and stand out from other types of APCs in their impact on immune tissue surveillance. DCs could be divided into immature cells (immature myeloid dendritic cells (iDCs)) and two groups of cells, which include matured cells (based on their origin): myeloid DCs (mDCs) and plasmacytoid DCs (pDCs) [1]. 

Many previous studies demonstrated the ability of monocytes to differentiate into mDCs in the presence of granulocyte-macrophage colony-stimulating factor (GM-CSF) and interleukin-4 (IL-4). It was proven that KG-1 (myelogenous CD34+) and THP-1 (monocytic) cell lines could easily differentiate into DCs [2]. In contrast, there were cell lines in those cytokines that could not promote differentiation of mDC precursors alone, for example, promyelocytic HL-60 or NB-4 cell lines. However, MUTZ (myelomonocytic) cell line has the highest potential to differentiate into mDCs. [3].

The knowledge regarding the impact of vitamin D on DCs significantly evolved since 1987, when the expression of vitamin D receptor (VDR) in DCs was reported for the first time.

The major progress in understanding how the active metabolite of vitamin D_3,_ 1,25(OH)_2_D_3_ (calcitriol) influences the antigen presentation in monocyte-derived DCs (mo-derived DCs) was made in 2000 by two independent research groups (Kumar and Adorini) who proved that calcitriol and its analogs inhibit the maturation of monocyte-derived DCs and suppress antigen presentation capacity. Both studies implied the role of vitamin D in the promotion of immune tolerance by inhibiting DC maturation and by an increase in the activity of suppressor/regulatory T cells (Tregs) [4,5,6].

Moreover, mature DCs show lower expression of VDR, compared with iDCs or monocytes, which means that they are relatively less sensitive to calcitriol. Moreover, high levels of 1,25(OH)_2_D_3_ synthesized by DCs enable cells to act on VDR-expressing iDCs, which, in turn, prevents them from further development [6].

The communication between DCs and T/B cells can be mediated by direct cell-to-cell contact, transfer of secreted molecules, or intercellular transfer of extracellular vesicles (EVs). The term “vesicles” includes microvesicles (MVs), ectosomes, and other microparticles (MPs). Initially, the term “exosome” (EX) was used for MPs ranging in size from 30 to 1000 nm. However, based on the origin, EXs refer to particles ranging from 30 to 100 nm, while MVs are larger in size and have a diameter of 100 to 1000 nm [7].

MVs are composed mainly of plasma membrane lipids and proteins and are released by shedding or budding from the surface of various cells. Their key role in the extracellular environment is related to altering, signaling, and facilitating cell invasion through cell-independent matrix proteolysis. Their ability to transfer proteins, DNA, mRNA, and miRNA facilitates the modification of the extracellular milieu and thereby the function of target cells [7,8].

Exosomes (EXs) derived from late endosomes are formed by inward budding of the limited multivesicular bodies (MVBs). Late endosomal membrane invagination results in the formation of intraluminal vesicles (ILVs) with large MVBs. This process is associated with the incorporation of proteins and their enclosing in ILVs that could be subsequently trafficked to lysosomes for degradation or released into the extracellular milieu [9].

Based on their origin, the composition of the EXs is complicated and includes GTPase Rab, SNAREs, annexins, flotillin, Alix1, and Tsg101, as well as the proteins associated with the lipid drafts. EXs are involved in the transport of various kinds of RNA, such as mRNA, miRNAs, snRNA, tRNA, vault RNA, Y RNA, and iRNA. All of them could be translated into proteins in recipient cells [10].

According to the WHO classification, based on the genetic abnormalities, acute B lymphoblastic leukemias (ALL-B), derived from precursor B cells, should be divided into two groups showing good or bad prognoses [11]. Patients with t(4;11)(q21;q23) *Mixed-Lineage Leukemia* (*MLL*) rearrangements belong to the poor-risk cytogenetic category, with overall disease-free survival (DFS) rate of about 25%, and produce an AF4/MLL fusion gene [12]. 

MLL gene encodes a DNA-binding protein, histone methyltransferase with *N*-terminal DNA-binding domain and C-terminal SET domain, and is usually found in infants leukemias (70%) or less frequently in older children suffering from leukemia (2–5%). The protein product of this gene is critical for embryonic development and hematopoiesis and has over 60 different fusion partners. The most known of them are AFF1 (AF4), MLLT3 (AF9), MLLT1, and MLLT10 (AF10) It is worth noting that each of them is related to a different prognosis; for example, the MLL-AF4, MLL-AF6, and MLL-AF10 are associated with bad prognosis, whereas MLL-AF9 is associated with the intermediate prognosis factor [13].

For many years, studies regarding the effects of vitamin D on B cells were limited, due to the lack of a direct impact on their maturation and proliferation, although active B cells are known to express the VDR receptors. In addition, the expression of the CYP27B1 enzyme in B cells indicates their potential for eliciting an autocrine/intracrine response to vitamin D analogs (VDAs) [6]. It was shown that calcitriol suppresses proliferation and immunoglobulin secretion due to the effects mediated by helper T cells (Th) and inhibits plasma cell differentiation and class-switched of the memory cells [6].

Therefore, we performed analyses to determine the impact of calcitriol and its analog tacalcitol on B cell proliferation, maturation, and function according to MLL translocation. We emphasized the difference between the direct effect of calcitriol and tacalcitol on B leukemic cells derived from ALL-B with MLL translocation, as well as B leukemic cells with mutations/translocations other than MLL and normal B cells, and indirect effects through EVs released from mDCs stimulated with calcitriol or tacalcitol. In this study, we used three commercially available acute B lymphoblastic leukemia cell lines with the t(4;11) translocation (SEM, KOPN-8, and RS4;11), one cell line (RCH-ACV) characterized by the presence of homozygotic deletion of CDKN2, which probably increases its proliferation ratio (t(11;19)), and normal B cells (LCL cell line) as a control. Detailed information on the origin of these cell lines is provided by Greil et al., Stong et al., and Jack et al. in their publications [14,15,16].

## 2. Materials and Methods

### 2.1. Cell Culture

A human monocyte cell line (SC) was obtained from American Type Culture Collection (ATCC, Rockville, MD, USA). Human acute B-lymphoblastic cell lines (KOPN-8, RS4;11, and RCH-ACV) and SEM were obtained from Leibniz Institute DSMZ-German Collection of Microorganisms and Cell Culture, Braunschweig, Germany. Normal EBV-immortalized B cell line (LCL) was generated in Viral Laboratory, Hirszfeld Institute of Immunology and Experimental Therapy, Polish Academy of Sciences, Wrocław, Poland. All cell lines were maintained at the Hirszfeld Institute of Immunology and Experimental Therapy, Polish Academy of Sciences, Wroclaw, Poland.

SC cell line was cultured in IMDM medium (Gibco, Scotland, UK) with 10% HyClone non-inactivated fetal bovine serum (FBS) (HyClone Laboratories, Logan, UT, USA), 50 × hypoxanthine–thymidine (HT) (Gibco, Scotland, UK) and B-mercaptoethanol (Sigma-Aldrich Chemie GmbH, Steinheim, Germany). iDCs were cultured in IMDM medium supplemented with GM-CSF and IL-4 (both from Gibco, Scotland, UK) for 7 days, with half of the medium being refreshed every 3 days (all from Gibco, Scotland, UK).

KOPN-8 and RCH-ACV cell lines, as well as normal B cells, were cultured in RPMI 1640 with GlutaMax medium (Gibco, Scotland, UK) supplemented with 10% FBS or 25% FBS in case of RCH-ACV (HyClone Laboratories, Logan, UT, USA). RS4;11 cell line was cultured in MEM-alpha medium with ribonucleosides and deoxyribonucleosides (Gibco, Scotland, UK) with 10% FBS (HyClone Laboratories, Logan, UT, USA) and SEM in IMDM with 10% FBS (HyClone Laboratories, Logan, UT, USA). All media were supplemented with 100 μg/mL of streptomycin (Sigma-Aldrich, Poznań, Poland) and 100 U/mL of penicillin (Polfa Tarchomin SA, Warsaw, Poland). Cells were cultured at 37 °C, in a 5% CO_2_ and humid atmosphere till the day of the next passage.

### 2.2. Compounds

Calcitriol (1,25(OH)_2_D_3_) and tacalcitol (1,24*R*(OH)_2_D_3_, PRI-2191) (both from Cayman Chemical, Ann Arbor, MI, USA) were dissolved in pure 98% ethanol (POCh, Gliwice, Poland) and stored in ampoules at −20 °C.

### 2.3. Anti-Proliferative In Vitro Test

Cells (1 × 10^4^/well) were seeded on 96-well plastic plates (Sarstedt, Numbrecht, Germany) in an appropriate culture medium. VDAs were dissolved in the test medium—OptiMEM (Gibco, Scotland, UK) and RPMI-1640 (IITD, PAN, Wrocław, Poland) (1:1), supplemented with 5% FBS (HyClone Laboratories, Logan, UT, USA), 2 mM L-glutamine (Polfa, Poznań, Poland), penicillin, and streptomycin—and added to the wells in triplicate (1000, 100, 10 and 1 nM). After 24, 48, 72, 96, 120, 144, and 168 h, the MTT test using [3-(4,5-dimethylthiazol-2-yl)-2,5-diphenyl tetrazolium bromide] was performed. Briefly, 20 μL of MTT (Sigma-Aldrich, Poznań, Poland) was added to all wells, and the plates were incubated at 37 °C, in a 5% CO_2,_ and humid atmosphere for 4 h. Then, plates were centrifuged at 800 rpm (88× *g*) for 5 min at 4 °C, and the supernatant was removed. Next, 200 μL of DMSO (POCh, Gliwice, Poland) was added to all test wells. The optical density values were assessed using an Elisa reader (BioTek, Synergy H4, Swindon, UK) equipped with Gen5 software. IC_50_ values (the dose which inhibits proliferation of 50% of the cell population) were estimated using Cheburator version 1.0.2 developed by Dmitry Nevozhay from the University of Texas MD Anderson Cancer Center, Houston, TX, USA [17]. The results are presented in the form of mean values ± SD. Each experiment was repeated at least 3 times.

### 2.4. Preparation of Immature Myeloid Dendritic Cells (iDCs)

The SC cell line was seeded at a density of 1 × 10^6^/3 mL in 6-well plates in IMDM complete medium, in the presence of 800 U/mL of recombinant human GM-CSF and 500 U/mL of human recombinant IL4 (both from Gibco, Scotland, UK), for 7 days. On days 3 and 5, one-half of the medium was replaced with fresh complete medium supplemented with cytokines. After 7 days, 10 nM of VDAs was added to iDCs. Finally, the cells were cultured in IMDM without FBS/BSA (bovine serum albumin) or its replacement for 96 h in a humid atmosphere and 5% CO_2_ before EV isolation.

### 2.5. Isolation of EVs

Immature myeloid dendritic cells were incubated with 10 nM of calcitriol or tacalcitol for 96 h. At the end of the incubation time, cells were collected and centrifuged at 300 g for 10 min at 4 °C (Sigma 3K-16, Newton, UK), to remove cell debris. The supernatant was collected and centrifuged at 2000× *g*, for 10 min at 4 °C, to remove fragments of cells. Again the supernatants were centrifuged at 10,000× *g* for 30 min at 4 °C (Ultracentrifuge, Sorvall VX+, Thermo Scientific, Waltham, MA, USA), to obtain MVs. Again, the supernatants were collected and centrifuged at 100,000× *g* for 70 min at 4 °C, to obtain EXs. Pellets were washed with large volumes of PBS pre-chilled to 4 °C to increase the purity of EXs. Pellets were resuspended in 100 μL of PBS and stored at −80 °C.

### 2.6. Total Protein Concentration

Total protein concentration was evaluated by the Bradford method according to the manufacturer’s instructions (BioRad, Herkules, CA, USA). The absorption was read using an Elisa reader (BioTek, Synergy H4, Swindon, UK) equipped with Gen5 software, at a wavelength of 650 nm.

### 2.7. Cytospin Staining

Cells (0.5 × 10^6^/mL of KOPN8, RS4;11, RCH-ACV, SEM, normal B cells (24 h/96 h), and DCs (96 h)) were seeded on a plastic TC Dish 60 (Sarstedt, Numrecht, Germany) with the addition of 10 nM VDAs. In the case of SC cell lines, cells were seeded on plastic plates at a density of 0.25 × 10^6^/mL (96 h). Next, samples were collected, washed with PBS containing 2% FBS (HyClone Laboratories, Logan, UT, USA) for 5 min, centrifuged at 1300 rpm (324× *g*) at 4 °C, and then added to a slide chamber SuperFrost (for May–Grunwald–Giemsa (MGG) staining) or SuperFrost Polylysine (for antigen staining) (both from Menzel-Glasser, Braunschweig, Germany) and centrifuged at 700× *g* for 7 min at RT (Shandon Cytospin 4, Thermo Scientific, Waltham, MA, USA). Prepared slides were kept at RT to dry completely.

For MGG staining, slides were fixed in ice-cold methanol (POCh, Gliwice, Poland) for 20 min and stained with May–Grunwald (Merck Millipore, Darmstadt, Germany) in Na/K buffer pH 7.2 (IITD PAN, Wroclaw, Poland) (1:1) solution for 5 min at RT, followed by staining with Giemsa (Merck Millipore, Darmstadt, Germany) in PBS pH 7.2 (1:9) solution for 15 min at RT. After the slides were completely dry, photographs were taken of slides under a microscope equipped with 20× and 100× immersion objective lens (Olympus model CX41, Olympus Europe Holding GMBH, Hamburg, Germany). All staining procedures were performed twice.

For antigen detection, slides were fixed with ice-cold acetone (POCh, Gliwice, Poland) for 20 min at RT, washed with TRIS-NaCl (IITD PAN, Wroclaw, Poland), and the endogenous peroxidase activity was quenched by incubating with Dako Dual Endogenous Systems (Dako Cytomation Poland, Gdynia, Poland) for 10 min at RT. Then, slides were incubated with 1:100 (HLA-DR) or 1:250 (anti-CD19 clone EPR5906) (both from Abcam, Cambridge, UK) for 1 h at RT. The complex of antigen-antibody was detected using Dako Real Envision Systems Peroxidase (Dako Cytomation Poland, Gdynia, Poland), according to the manufacturer’s instructions. Nuclei counterstaining was performed using Gill’s III hematoxylin (Merck Millipore, Darmstadt, Germany). All staining procedures were performed twice.

Microscopic analysis was performed and photographs were captured under a bright field microscope (Olympus model CX41, Olympus Europe Holding GMBH, Hamburg, Germany), using a camera equipped with Olympus Stream Image Analysis Software. Microscopic examination was performed using 20× and 100× immersion objective lens, and slides were graded according to staining intensity as follows: lack of staining—0; faint staining—1+; intermediate staining—2+; intense staining—3+; right staining—4+.

### 2.8. Flow Cytometry Analysis of Apoptosis

Cells (0.25 × 10^6^/well) were seeded on 24-well plastic plates (Sarstedt, Numbrecht, Germany) in an appropriate culture medium. VDAs were dissolved in the test medium, as described in “Section 2.3. Anti-Proliferative In Vitro Test”. After 96 h of incubation, SC cells and iDCs cells derived from them were collected and centrifuged at 324× *g* for 5 min at 4 °C (Sigma 3–16K, Newton, UK), washed with PBS for 5 min, and finally resuspended in annexin binding buffer (IIET, PAS, Wroclaw, Poland), at the density of 5 × 10^5^ cells/mL. The cells were stained with APC-conjugated annexin V for 15 min, at RT, in the dark, and washed with PBS for 5 min at 324× *g*. Pellets were resuspended in annexin-binding buffer, and PI (propidium iodide) (Merck KGaA, Darmstadt, Germany) at a concentration of 50 μg/mL was added to each sample. At least 20,000 cells were collected, and the percentage of cells in the early apoptosis, late apoptosis, and necrosis were read using BD LSR Fortessa II (Becton Dickinson, Franklin Lakes, NJ, USA) equipped with FACS Diva 6.1. Software. The results were analyzed using Flowing Software version 2.5.1, developed by Pertho Tertho from Turkey. Each experiment was performed three times independently.

### 2.9. Cell Cycle Analysis

Cells (0.25 × 10^6^/well) were seeded on 24-well plastic plates (Sarstedt, Numbrecht, Germany) in an appropriate culture medium. VDAs were dissolved in the test medium, as described in “Section 2.3. Anti-Proliferative In Vitro Test”. After 96 h of incubation, SC cells and iDCs cells derived from them were collected and centrifuged at 324× *g* for 5 min at 4 °C (Sigma 3–16K, Newton, UK). Next, samples were washed twice with PBS for 5 min, centrifuged at 324× *g* at 4 °C, resuspended in 70% ethanol in distilled water (POCH, Gliwice, Poland), and stored at −20 °C for at least 24 h.

For the analysis of the percentage of cells in the particular cell cycle phases, thawed pellets were washed with PBS and incubated with a solution of RNAse A, DNAse, and protease-free PBS (Life Technologies, Waltham, MA, USA) at a concentration of 8 μg/mL for 1 h at 37 °C, with gentle shaking. Just before flow cytometry reading, PI was added to all samples. At least 10,000 events were collected, and the content of the cellular DNA was read using BD LSR Fortessa II (Becton Dickinson, Franklin Lakes, NJ, USA) equipped with FACS Diva 6.1. software. The percentage of the cells in the particular cell cycle phases was assessed by Flowing Software version 2.5.1 developed by Pertho Tertho and by ModFiT Software version 3.2. (Verity Software House, Topsham, UK). Each experiment was performed three times independently.

### 2.10. Flow Cytometry Analysis

Cells in 4 wells (0.25 × 10^6^/well) were incubated with 10 nM calcitriol and tacalcitol for 24/96 h, collected, centrifuged, and washed with PBS (IITD PAN, Wrocław, Poland) containing 2% FBS (HyClone Laboratories, Logan, UT, USA) for 5 min, centrifuged at 324× *g*, at 4 °C. The total cell number was assessed using Trypan blue staining (Merck KGaA, Darmstadt, Germany). Each sample was divided into a control and test probe and stained with a primary antibody (Table 1) (all from Becton Dickinson, Franklin Lakes, NJ, USA) for 30 min in the dark at RT, washed with PBS, and read out using BD LSR II Fortessa (Becton Dickinson, Franklin Lakes, NJ, USA) equipped with Diva 6.0. software. At least 30,000 cells were read, and the analysis was performed using Flowing Software version 2.5.1. developed by Perthu Tertho from Turkey.

In experiments with the cells (SC, iDCs, and B normal/leukemia cells), the R1 gate was set up on FSC/SSC in the linear scale on the unstained samples (auto-control). The R1 gate was corrected after SSC/PI gating of live cells. The percentage of positive cells was read in gate H2 obtained from the overlaid histograms for specific markers, according to auto-control samples.

In the experiments with the EVs, the R1 gate was set up on the FSC/SSC logarithmic scale. The percentage of the positive cells was read in gate H2 obtained from the overlaid histograms for specific markers, according to auto-control samples. The compensation was calculated automatically using the FACS Diva 6.0 software.

Exosome samples (5 μg/sample) were incubated with 4 μm diameter aldehyde–sulfate latex beads (Thermo Scientific, Waltham, MA, USA) for 15 min at RT. Next, the samples were incubated for 24 h at 4 °C with gentle rotation. After adding 1M glycine (final concentration 100 mM) (Sigma-Aldrich Chemie, Poznań, Poland) samples were kept at RT for 30 min and centrifuged at 4000 rpm (3076× *g*) for 3 min at RT, and pellets were resuspended in PBS with 0.5% BSA (Sigma-Aldrich Chemie GmbH, Poznań, Poland). Then, EXs were incubated with the primary antibodies (Table 1), washed and resuspended in PBS with 0.5% BSA, and read using BD LSR II Fortessa (Becton Dickinson, Franklin Lakes, NJ, USA) equipped with Diva 6.0. software. At least 50,000 events were read, and the analysis was performed using Flowing Software version 2.5.1., developed by Perthu Tertho from Turkey. Each experiment was repeated at least 3 times.

For the phenotypic characterization of MVs, 5 µg/sample of MVs was incubated with PBS with 0.5% BSA for 30 min at RT, and then an appropriate antibody was added to each sample and incubated for 20 min at RT in the dark. Finally, probes were read using BD LSR II Fortessa (Becton Dickinson, Franklin Lakes, NJ, USA) equipped with Diva 6.0. software. At least 50,000 events were read, and the analysis was performed using Flowing Software version 2.5.1. developed by Perthu Tertho from Turkey [18]. Each experiment was repeated at least 3 times.

### 2.11. Culturing of Short-Term ALL-B Precursor Cells with the Addition of EVs

According to the previous studies [19], we performed a short-term culture (24 h) study using 10 μg of EVs and B cells. Cells were cultured at the 37 °C, and afterward, the supernatant was collected and frozen at −80 °C; then, we analyzed IgM and IgG (the protocol to detect IgM and IgG by using an ELISA reader is given in Section 2.12), while the pellets were washed with PBS and 0.5% BSA and analyzed by flow cytometry, as described in “Section 2.10. Flow Cytometry Analysis”. Each experiment was performed at least three times. 

### 2.12. Assessment of MVs/EXs Hydrodynamic Size via DLS (Dynamic Light Scattering)

The hydrodynamic size of MPs was determined using Zetasizer Nano ZS (Malvern, Worcestershire, UK), with the following parameters: refractive index (1.450) and solvent viscosity (PBS, 0.9060 10^−4^ Pa×s).

About three-to-six consecutive measurements of each sample were taken, with an acquisition time of 30 sec per correlation function. Data were analyzed using DTS 6.10 software (Malvern Instruments, Worcestershire, UK). Particle-size distributions were obtained using the general-purpose algorithm included in the DTS software. Each sample was prepared independently 3–4 times from the same sample as flow cytometry phenotyping was performed. All functional and further statistical analyses were performed in monodispersed samples with a polydispersity index (PdI) <0.400.

### 2.13. Biological Nanostructure Imaging by Cryogenic Low-Voltage Field-Emission Scanning Electron Microscopy (Cryo-LV-FESEM)

For imaging of MPs released from cultured iDCs, Cryo-EM with an adopted low-voltage (LV) mode was used. Due to the low energies of incident beam electrons (800 eV), sufficient image contrast was generated directly from the endogenous components of biological samples consisting mostly of low atomic number (Z) elements, surpassing the need to coat with heavy metals to enhance the contrast [20]. The electron density of low atomic numbers (Z) elements such as C, H, O, S, N, and P are low, so LV-FESEM offers the unique property of evading such contrast and allows for direct observation of biological objects in their native composition. Vitrification of the sample (under high pressure or by slushing/freezing) allows for the observance of natively hydrated states despite a high vacuum in the scanning electron microscope chamber. The coating-free approach within Cryo-LV-FESEM mode, which omits extraction, allows for analysis of MVs/EXs based on the topography of frozen and fractured natively hydrated samples. The details of topography/texture were observed at high resolution, compatible with the expected dimensions of individual vesicles. Vitrifically frozen samples under high vacuum (10^−5^ mBar) and at low temperature (−140 °C) fracture preferentially at lipid–bilayer interfaces rather than across hydrated volume; thus, lipid-bilayer-sequestered vesicles are efficiently represented on fracture-exposed surfaces.

The suspension of MVs/EXs in PBS was deposited as a 50 µm droplet on the polished silicon crystal (100), and the technique of slush-nitrogen vitrification was applied at −210 °C. The cryo-vitrified suspension was observed immediately upon fracturing, under low energies of electron beam and at −140 °C, with SE2 electrons detected with Everhart-Thornley (ET) detector (Auriga 60, Carl-Zeiss Oberkochen, Germany equipped with Quorum Technology cryo-stage and preparative chamber PP3010), as described previously [21].

### 2.14. IgM and IgG Determination in Precursor B Cells

Quantitative detection of human IgM/IgG in supernatants was performed according to the manufacturer’s instructions (Human IgM/IgG Platinum Elisa eBioscience, Thermo Scientific, Waltman, MA, USA). The absorbance was read on a plate reader using 450 nm as the primary wavelength and optionally 620 nm as the reference wavelength. The average absorbance was calculated for standards and samples, and the concentration of circulating human Ig was determined according to the standard curve obtained by plotting the mean absorbance for each standard concentration. Each sample from three independently collected supernatants was analyzed in duplicate using ELISA tests.

## 3. Results

### 3.1. The Sensitivity of SC and iDCs on Calcitriol and Tacalcitol

**Figure 1:**
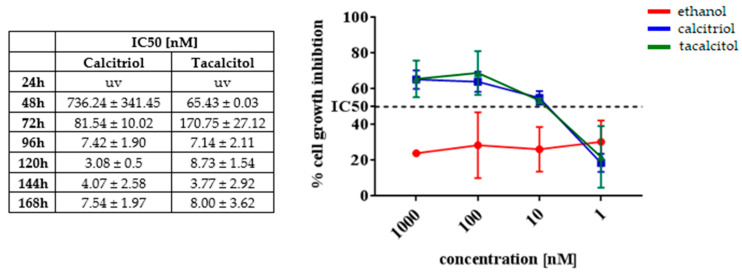
Kinetics of anti-proliferative effects of vitamin D analogs against SC cell lines. Table presents IC_50_ values calculated after different periods of incubation. Graph presents kinetics of inhibition of SC cell line proliferation in various concentrations of calcitriol and tacalcitol after 96 h of incubation. Mean values and standard deviation are presented. Each measurement of anti-cytotoxicity effects (at each time point and at each concentration) was performed at least 3 times on independently seeded cells). Abbreviation: uv, undetermined.

To study the impact of calcitriol and tacalcitol on SC cell lines and iDCs derived from them, an MTT test was performed. Subsequently, changes observed in the kinetics during culture were analyzed. The results were estimated as IC_50_ values—the concentration that inhibited 50% of cell growth (Figure 1).

According to earlier studies, monocytes [22,23,24] and mDCs obtained from them [25,26,27,28] were sensitive to the analogs of calcitriol. We were able to determine the IC_50_ value after 48 h of stimulation; however, the best results were obtained after 96 h, and based on these findings, a time period of 96 h and 10 nM concentration were chosen for further experiments. At none of the analyzed time points did ethanol cause any impact on the final results. Results for SC cell line are presented in Figure 1.

To confirm the sensitivity of the obtained iDCs to calcitriol and tacalcitol, an MTT test was performed for 96 h, and IC_50_ values were calculated as 5.69 ± 0.83 nM and 1.56 ± 0.63 nM, respectively.

Additionally, based on the translocation of phosphatidylserine to the outer layer of the plasma membrane the discrimination between early apoptosis from late apoptosis and necrosis upon VDA stimulation was performed. The results indicate that both VDAs could decrease the percentage of live cells while increasing the percentage of necrosis of parental cells. In turn, in iDCs only calcitriol increased the percentage of late apoptotic cells (Supplementary File Figure S1).

The impact of VDAs on the distribution of cells in the cell cycle was observed in parental and generated cells. In SC cell line, the percentage of cells in the S phase was decreased after calcitriol and tacalcitol stimulation, while in iDC stimulation, with an increase in calcitriol, the percentage of cells in the sub-G0 phase increased (Supplementary File Figure S2).

### 3.2. Enhanced Vacuolization in Calcitriol- and Tacalcitol-Stimulated Myeloid iDC/Mat DC Cells

**Figure 2:**
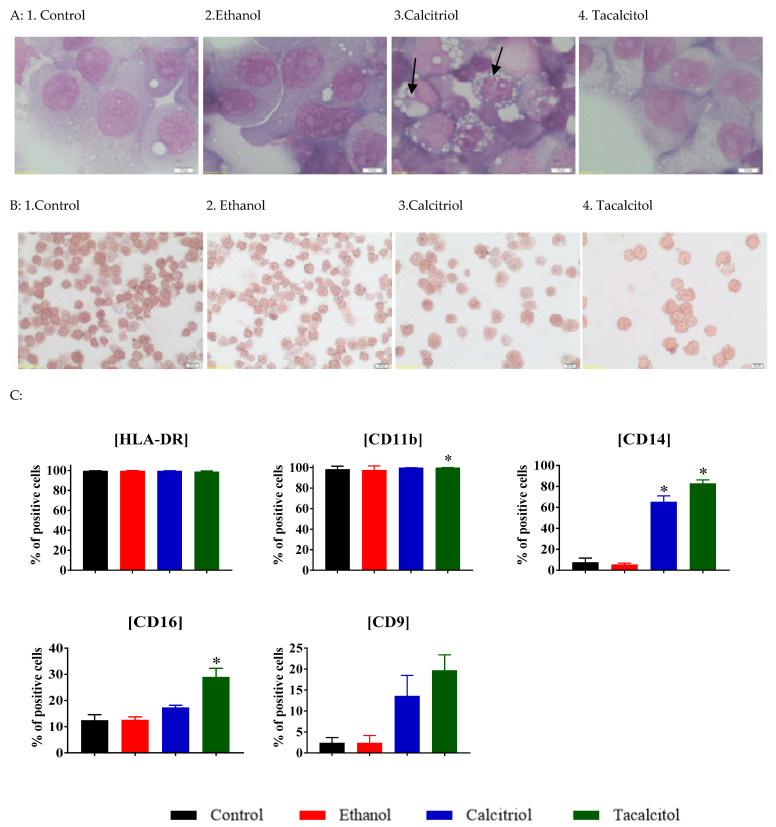
Morphology and differentiation of parental monocytes (SC cell lines) upon calcitriol and tacalcitol treatment: (**A**) May–Grunwald–Giemsa staining scale bar 10 μm. Each staining was performed independently twice; Black arrows indicate the vacuolization inside the cells. (**B**) immunocytochemical HLA-DR staining scale bar 20 μm. Each staining was performed independently twice; (**C**) distribution of differentiation markers by flow cytometry analysis. Mean values and standard deviation are presented. Statistical analysis: Sidak’s multiple comparisons test; significant differences as compared with ethanol (* *p* < 0.005). The graphs represent data from at least three separate experiments. Blank arrow indicate the vacuolization inside the cells.

Morphologically, our monocytes/macrophages were large cells, with a medium-size nucleus and clearly visible nucleoli (one or two) in all samples. In the abundant cytoplasm, a few small or single large vacuoles were visible. Strong vacuolization (presence of abundant small vacuoles) was observed in calcitriol- and tacalcitol-stimulated cells (Figure 2A).

Cells were positive for HLA-DR immunocytochemical staining. The membranes of all cells showed a staining intensity of about 2+/3+, but there were no differences in the staining intensity between control and calcitriol- or tacalcitol-stimulated cells (Figure 2B).

To characterize and describe how cell culture with the addition of calcitriol and tacalcitol influences monocyte formation, we performed a preliminary phenotypic analysis. For this purpose, we analyzed the expression of HLA-DR, CD9, CD11b, CD14, and CD16. A statistically significant increase in the CD14 expression was observed for calcitriol, compared with ethanol control, and for tacalcitol, an increase in expression was observed for CD11b, CD14, and CD16 markers, which might probably be the result of monocyte differentiation (Figure 2C); however, for CD11b, the effect had limited biological significance (the difference was less than twofold). This finding was compatible with our previous results [29].

Although cells, seemed to show an increasing tendency, as observed in Figure 2C CD9, the results were not statistically significant (ethanol control vs. calcitriol (*p* = 0.1420) and ethanol control vs. tacalcitol (* *p* = 0.0591) in Sidak’s multiple comparison test).

**Figure 3:**
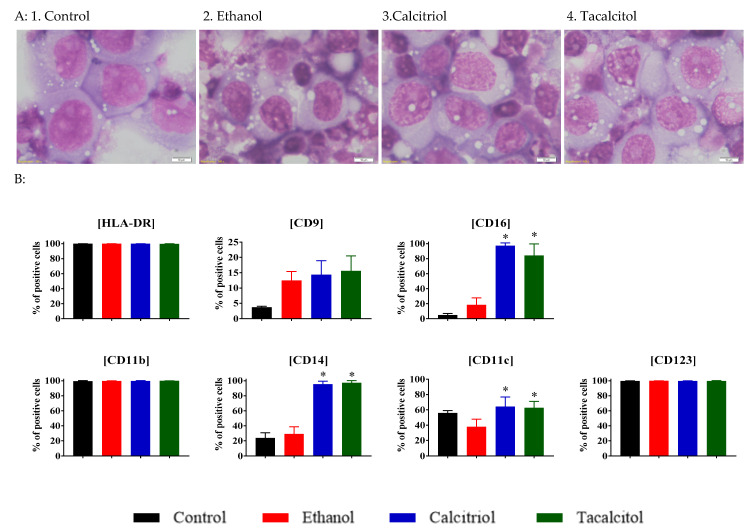
Morphology and differentiation of myeloid-derived iDCs upon calcitriol and tacalcitol treatment: (**A**) May–Grunwald–Giemsa staining; scale bar 10 μm. Each staining was performed independently twice; (**B**) distribution of differentiation markers by flow cytometry analysis. Mean values and standard deviation are presented. Statistical analysis: Sidak’s multiple comparisons test; significant differences as compared with ethanol (* *p* < 0.005). The graphs represent data from at least three separate experiments.

Untreated myeloid-iDCs were large cells with nuclei characterized by the presence of three or four clearly visible nucleoli (Figure 3A). In myeloid-iDCs, we observed the same effect of intensification of vacuolization after calcitriol/tacalcitol treatment, although the vacuoles were larger than those in control samples. Interestingly, the chromatin structure of the nuclei of iDCs treated with calcitriol and tacalcitol was loose, which is typical for blasts (Figure 3A).

For phenotyping of iDCs (Figure 3B) we analyzed the expression of HLA-DR, CD11c, CD123, CD9, CD16, and additionally CD11b and CD14 because their expression was expected to change following stimulation by VDAs. The percentage of HLA-DR, CD11b, and CD123 positive cells were stable after calcitriol or tacalcitol treatment. As compared with ethanol control, we observed a statistically significant increase in the percentage of CD11c, CD14, and CD16 (Figure 3B).

### 3.3. Phenotype Differentiation of EVs Derived from SC/iDCs after Calcitriol and Tacalcitol Treatment

According to previously reported results about MVs and EXs, we selected the markers for their determination (Table 1). For EXs, surface tetraspanins are recommended for assessing the origin of MPs [30]. From among a variety of cell surface membrane proteins, we chose CD9, CD63, and CD81 as markers.

**Figure 4:**
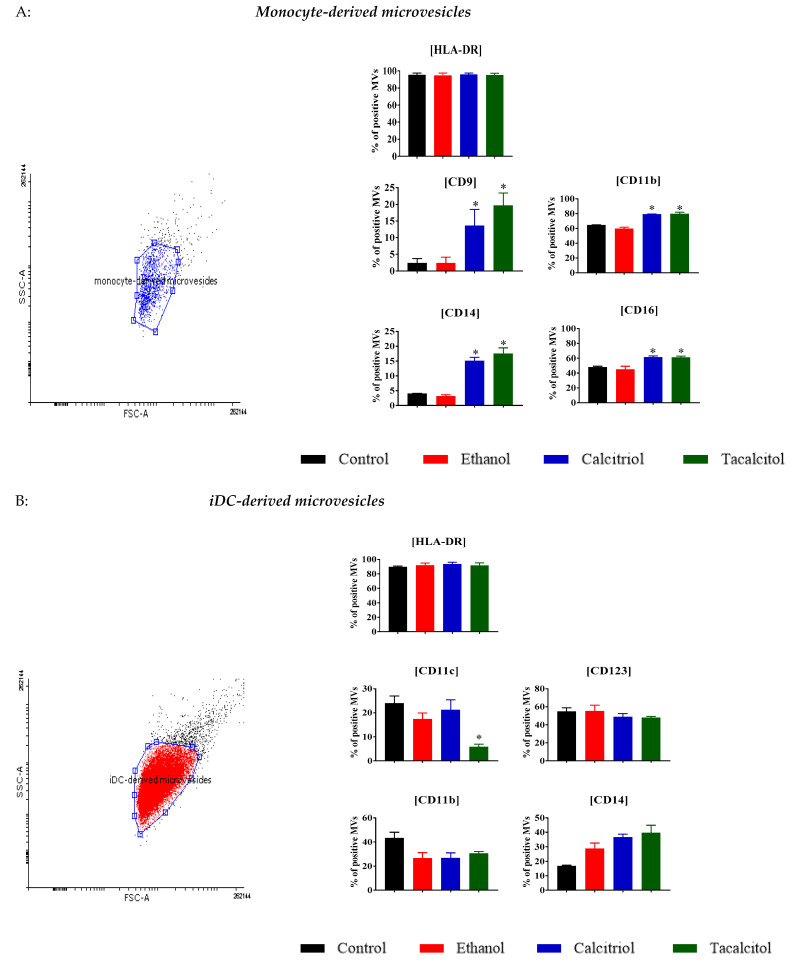
Characteristics of monocyte-derived and myeloid iDC-derived MVs: (**A**) distribution of differentiation markers of monocytes-derived MVs by flow cytometry analysis. Mean values and standard deviation are presented. Statistical analysis: Sidak’s multiple comparisons test; significant differences as compared with ethanol (* *p* < 0.005). The graphs represent data from at least three separate experiments; (**B**) distribution of differentiation markers of myeloid iDCs-derived MVs by flow cytometry analysis. Mean values and standard deviation are presented. Statistical analysis: Sidak’s multiple comparisons test; significant differences as compared with ethanol (* *p* < 0.005). The graphs represent data from at least three separate experiments.

In our experiments, we analyzed the percentage of positivity of HLA-DR, CD9, CD11b, CD14, and CD16 for parental cell-derived MVs (Figure 4A), and HLA-DR, CD11c, and CD123 for DC-derived MVs to confirm their origin. We also studied positive expressions of CD11b and CD14, as they are useful markers for the determination of the percentage of monocyte/myeloid cells that differentiated after VDA treatment (Figure 4B). We noticed that the MVs expressed the same surface markers as those of the cell of origin and that the VDAs could cause an impact on the phenotype of MVs.

In monocyte-derived MVs, an increase was observed between ethanol control vs. calcitriol and tacalcitol in the percentages of CD9, CD11b, CD14, and CD16, which, in the case of CD14 and CD16, indicated the impact of VDAs on monocyte cells (Figure 4A).

In the MVs obtained from iDCs, we observed that the vast majority of MVs were HLA-DR positive. The percentage of positive CD11c MVs was decreased after tacalcitol treatment in comparison with MVs derived from ethanol-stimulated cells (Figure 4B).

**Figure 5:**
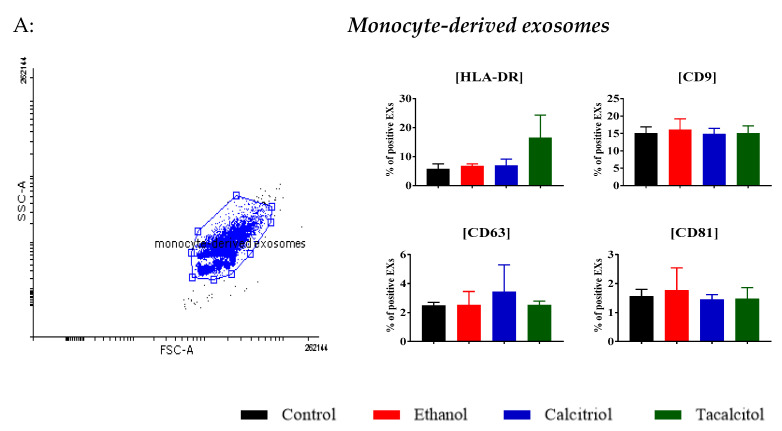

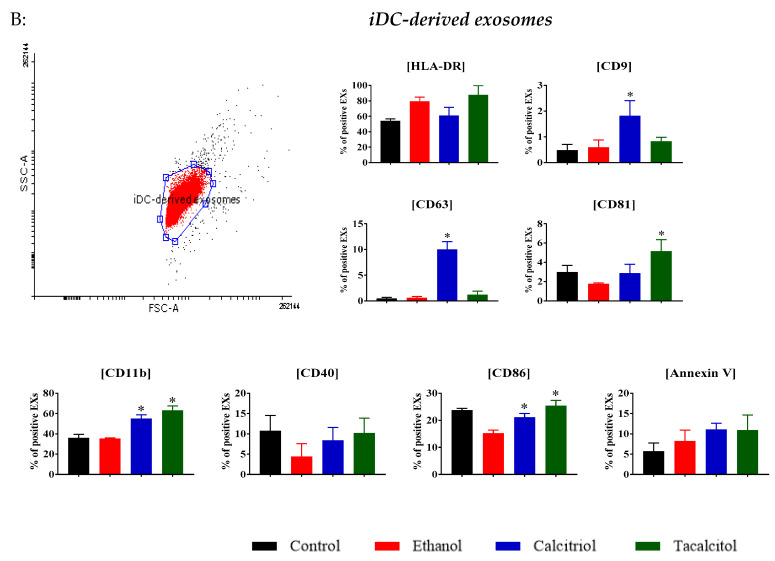
Characteristics of monocyte-derived and myeloid iDC-derived EXs: (**A**) distribution of differentiation markers of mo-derived EXs by flow cytometry analysis. Mean values and standard deviation are presented. Statistical analysis: Sidak’s multiple comparisons test; significant differences as compared with ethanol (* *p* < 0.005) The graphs represent data from at least three separate experiments; (**B**) distribution of differentiation markers of myeloid iDC-derived EXs by flow cytometry analysis. Mean values and standard deviation are presented. Statistical analysis: Sidak’s multiple comparisons test; significant differences as compared with ethanol (* *p* < 0.005). The graphs represent data from at least three separate experiments.

Similarly, EXs derived from parental cells (SC cell line) differed from those produced by myeloid DCs (Figure 5A,B). In monocyte-derived EXs, the percentage of HLA-DR positive EXs was lower than that observed in iDCs. The percentages of CD9- and CD63-positive EXs were increased in calcitriol-stimulated iDC samples. In turn, the percentage of CD81 positive EXs was increased in tacalcitol-stimulated iDC samples. Additionally, both vitamin D analogs increased the percentage of CD11b (adhesion molecule)- and CD86 (immunoregulatory molecules)-positive EXs derived from iDCs (Figure 5B).

**Figure 6:**
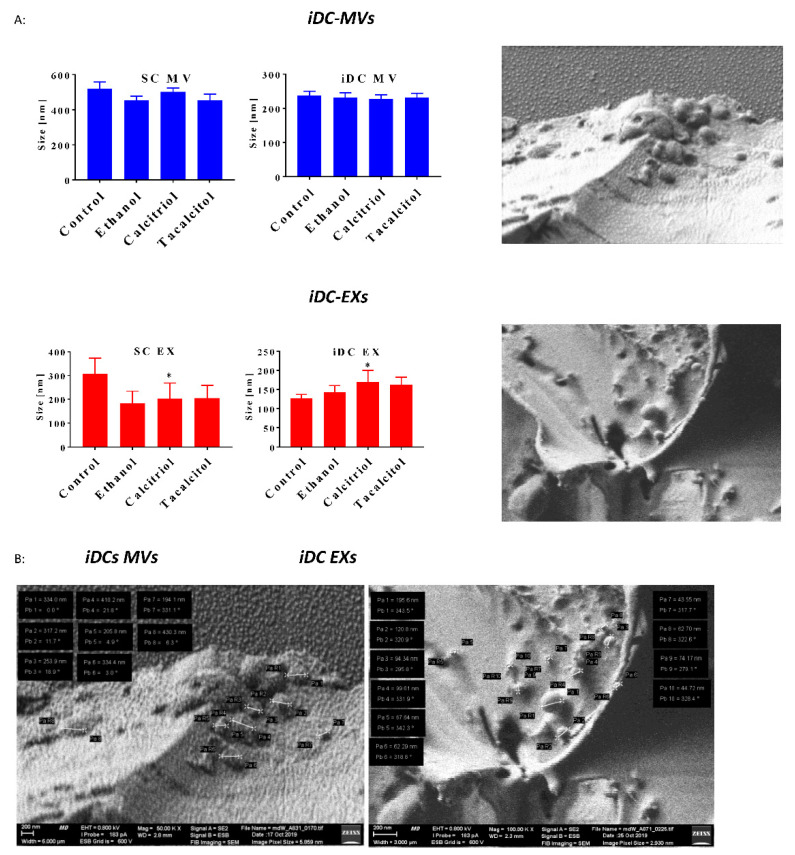
Size distribution of microvesicles (MVs) and exosomes (EXs) derived from parental monocytes and myeloid-derived iDCs: (**A**) size distribution of MVs/EXs-derived from parental cells/iDCs. Mean values and standard deviation are presented. Statistical analysis: Sidak’s multiple comparisons tests were performed. Significant differences compared with ethanol are marked in the figure; (* *p* < 0.005). The graphs represent data from the three-to-six consecutive measurements of at least three separate collected samples taken with an acquisition time of 30 sec per correlation function; (**B**) freeze–fracture distribution of MVs/EXs derived from iDCs (control). iDCs-derived MVs (control): magnification, 50,000×; field of view, 6 µm; iDCs-derived EXs (control): magnification, 100,000×, field of view, 3 µm).

The size and distribution of microparticles, known as dispersity, were assessed using Zetasizer Nano S, which employs a 173° backscatter detector. The size and distribution of MPs are presented as mean size (nm), standard deviation (SD), and polydispersity index (PdI), and the results indicate variations in the particle size [31,32] (Figure 6A). After the analysis, we rejected the values in which the PdI was more than 0.400. We observed that MPs obtained from monocytes were almost twice as large as those derived from myeloid iDCs (Figure 6A,B). In the case of MVs, the mean size of particles derived from monocytes oscillated around 450–550 nm, and those from the myeloid-derived iDCs were smaller in size (about 250 nm) (Figure 6A). EXs also showed a similar trend—namely, particles from monocytes were larger than those obtained from myeloid iDCs.

Additionally, we observed a statistically significant increase in the size of EXs derived from calcitriol-treated monocytes and myeloid iDCs, compared with ethanol control (Figure 6A).

In Cryo-LV-FESEM imaging, we observed multiple vesicles of various sizes and dimensions in the control samples (Figure 6B). The size of the obtained MPs corresponded to the results obtained from DLS.

### 3.4. Morphology and Phenotype Changes of Acute Lymphoblastic Precursor B Cells after Calcitriol or Tacalcitol Treatment

**Figure 7:**
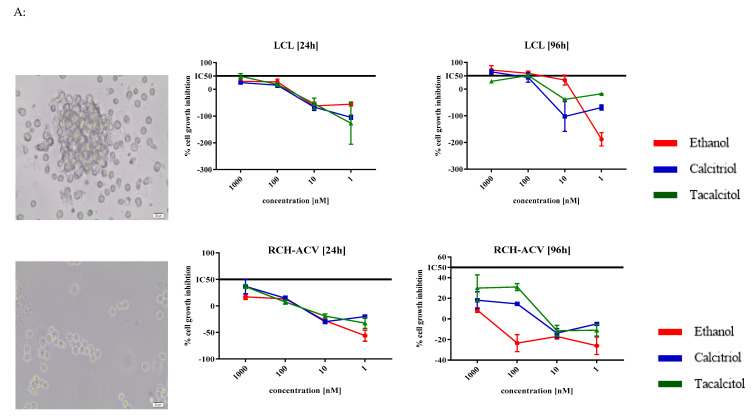

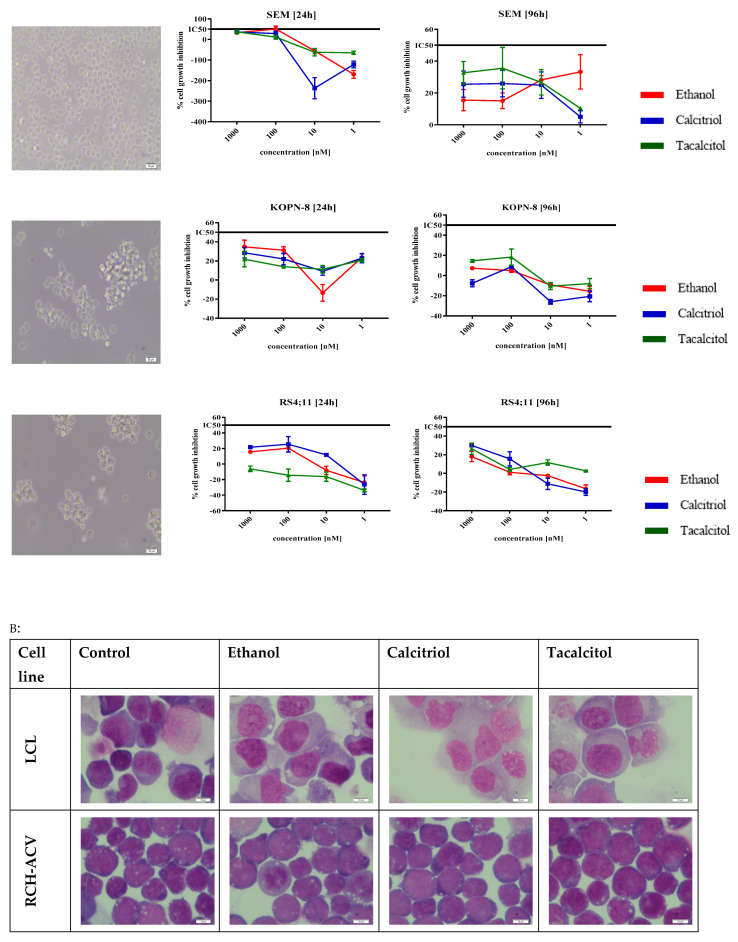

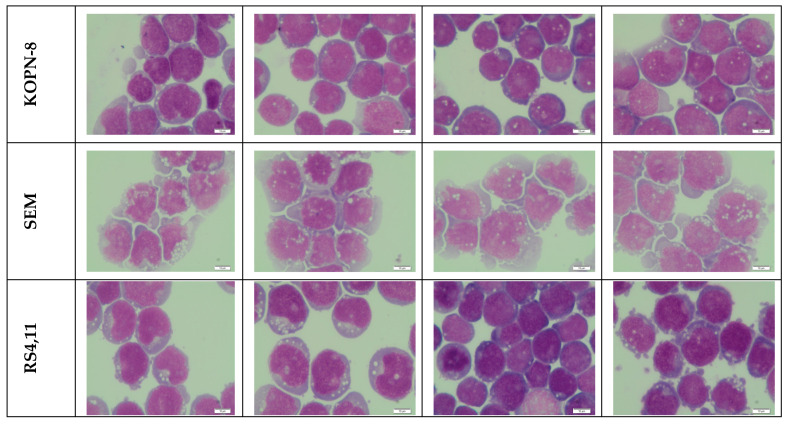
Morphology and kinetics of growth inhibition effect of calcitriol and tacalcitol against B lymphocytes after 24 h/96 h. Notes: (**A**) cluster of cells observed under the inverted microscope with 20× objective (scale bar 20 μm). Each photograph was captured from two independent repeats. Kinetics of anti-proliferative effects of vitamin D analogs against B lymphocytes were analyzed. Mean values and standard deviation are presented. Each measurement of anti-cytotoxicity effects (at each time point and at each concentration) was performed at least 3 times on independently seeded cells; (**B**) May–Grunwald–Giemsa staining scale bar 10 μm. Each staining was performed twice.

**Figure 8:**
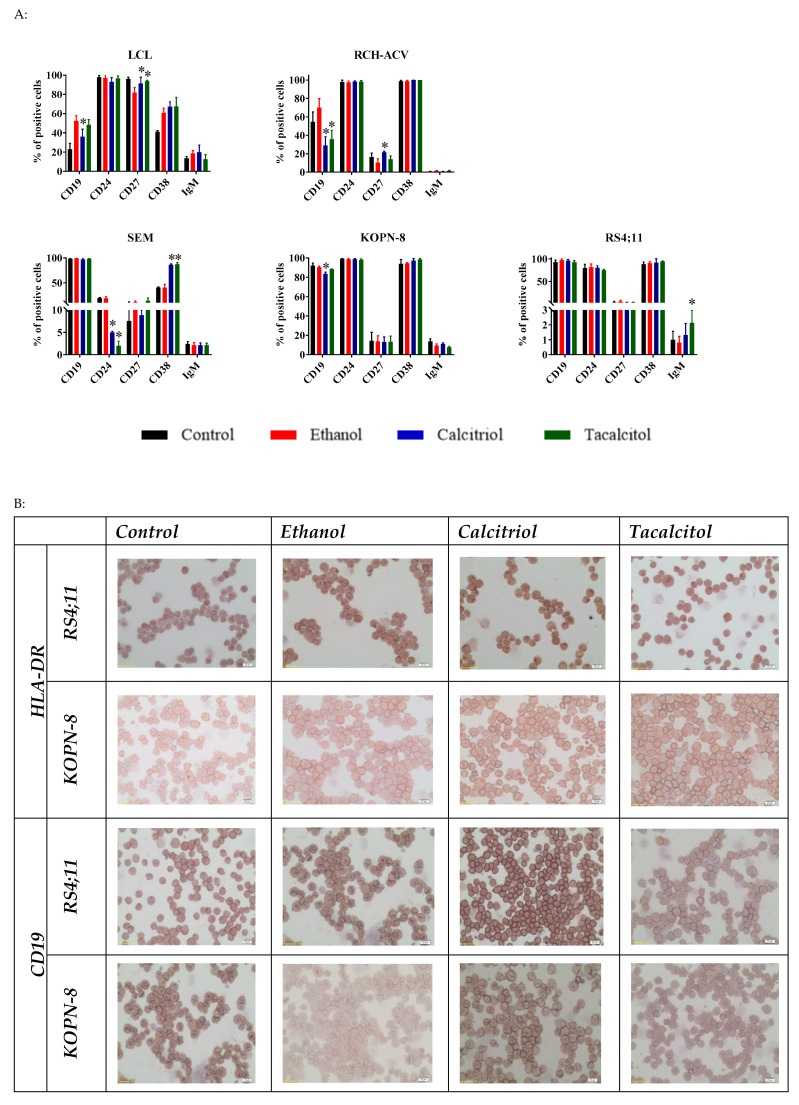
The direct effects of calcitriol and tacalcitol on the distribution of CD markers on normal and leukemic B cells. Cells were incubated for 96 h with 10 nM of calcitriol and tacalcitol or 1% ethanol: (**A**) flow cytometry distribution of CD markers. Mean values and standard deviation are presented. Statistical analysis: Sidak’s multiple comparisons test; significant differences as compared with ethanol (* *p* < 0.005). The graphs represent data from at least three separate experiments; (**B**) localization of HLA-DR and CD19 in B leukemic cells. Scale bar 10 µm. Each staining was performed at least twice.

Cell lines were analyzed according to the direct impact of calcitriol and tacalcitol on their morphology, kinetics of growth inhibition (Figure 7A,B), and phenotype (Figure 8A,B).

Firstly, we observed the morphology of the cells under an inverted microscope and analyzed the kinetics of growth inhibition after incubation with calcitriol and tacalcitol. Each cell line was characterized by rather small near-round cells that grow in suspension and form clusters except for the SEM cell line (Figure 7A).

The proliferation of almost all cell lines was not significantly affected by the treatment when compared with ethanol control. Only the RCH-ACV cell line was found to be inhibited by 20–30% after 96 h of incubation with 10 nM calcitriol and tacalcitol. Moreover, stimulation of SEM cell line proliferation was observed after 24 h of incubation with 10 nM tacalcitol and to a lesser extent with 1 nM concentration of both compounds. The results for 24 h and 96 h are presented in Figure 7A, and the results from other time points are presented in Supplementary Files (Figures S3–S7).

Additionally, we performed morphology analysis using May–Grunwald-Giemsa staining to investigate the effect of VDAs on B cells. We used a 10 nM concentration of calcitriol or tacalcitol and compared changes in the morphology of cells after 96 h of incubation (Figure 7B).

Morphologically, the LCL cell line resembled the plasma cells and exhibited a small, rather dense, and eccentric nucleus. The cytoplasm was abundant with occasionally visible vacuoles. In turn, the RCH-ACV cell line was similar to B lymphoblasts and was characterized by a large, delicate nucleus, and rarely visible nucleoli. The cytoplasm was dark blue (basophilic) with small vacuoles, which confirmed the blast stadium of B cell development. Morphologically, the KOPN-8 cell line seemed to be older and had a large nucleus of various shapes with delicate structure, single nucleolus, and a small amount of dark-blue cytoplasm with a single vacuole, while the SEM cell line had a delicate nucleus with more than one nucleolus; in addition, the cytoplasm was dark blue, and the vacuolization was clearer than in other cell lines. The cells were characterized by pleomorphism in size and structure. In the RS4;11 cell line, a large nucleus occurred in various shapes and had more than one nucleolus, and the cytoplasm was blue with small vacuoles and a bright hallo. Stimulation with calcitriol and tacalcitol did not cause any effect on the morphology of the leukemic B cells, except for the mild intensity of vacuolization in the tacalcitol-treated KOPN-8 cell line. We also observed that normal B cells treated with VDAs seemed to be slightly larger in size (Figure 7B).

We used the following antigens for the phenotyping of B cells: HLA-DR, CD9, CD10, CD19, CD20, CD23, CD24, CD27, CD34, and CD38, as well as surface IgM and IgD. We compared the phenotype of B cells after 96 h of stimulation with VDAs to characterize their impact on the distribution of differentiation markers. The results are presented and summarized in Figure 8A and in Table S1 of Supplementary Files.

We observed statistically significant impacts of calcitriol and tacalcitol on CD19, CD24, CD27, CD38, and IgM expression. VDAs caused the increase in CD27 (LCL and RCH-ACV), CD38 (SEM), and IgM (RS4;11) expression, while a marked decrease was seen in the case of CD19 (LCL, RCH-ACV, and KOPN-8) and CD24 (SEM) (Figure 8A) expressions. We observed that LCL (normal B cells) were completely phenotypically different from leukemic B cells. They were characterized by an increased percentage of CD10, CD20, CD23, CD27, CD34, and IgM, while the expression of CD19 was lower than that observed in leukemic B cells, independently of the stimulation with VDAs (Figure 8A and Supplementary Files Table S1).

The immunocytochemical staining confirmed the presence of CD19 and HLA-DR in calcitriol- and tacalcitol-stimulated cells (Figure 8B). Here, we present the results for RS4;11 cell line, for which the flow cytometry results showed that the expression of HLA-DR oscillated around 60%, and for KOPN-8 and other cell lines, for which HLA-DR expression oscillated around 100%. In all cases, we observed HLA-DR- and CD19-positive cell membrane staining. The intensity of HLA-DR in both cell lines and CD19 in the RS4;11 cell line was about 3+ (Figure 8B).

### 3.5. Morphology and Phenotype of Precursor B Cells Cultured with EVs Derived from Myeloid iDCs Treated with Calcitriol and Tacalcitol

**Figure 9:**
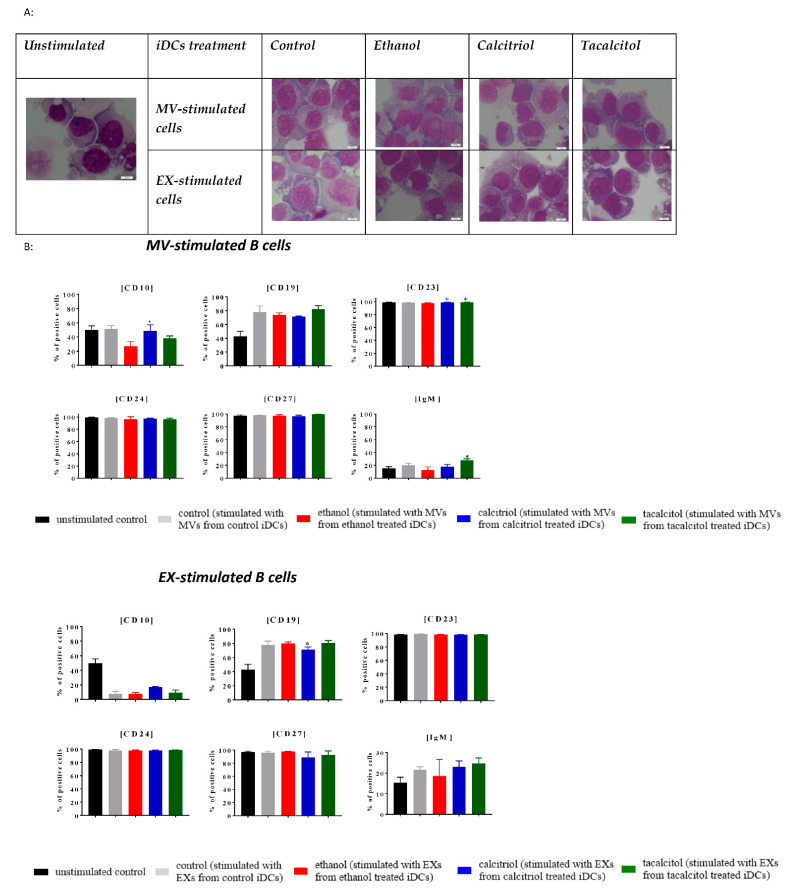
The indirect effects of calcitriol and tacalcitol on LCL cell line after 24 h of stimulation with EVs derived from iDCs: (**A**) morphology of EV-stimulated LCL cell line; scale bar 10 μm. Each staining was independently performed twice; (**B**) distribution of differentiation markers by flow cytometry analysis. Mean values and standard deviation are presented. Statistical analysis: Sidak’s multiple comparison tests in comparison with ethanol were performed; significant differences as compared with ethanol (* *p* < 0.005). Each experiment was performed at least 3 times.

**Figure 10:**
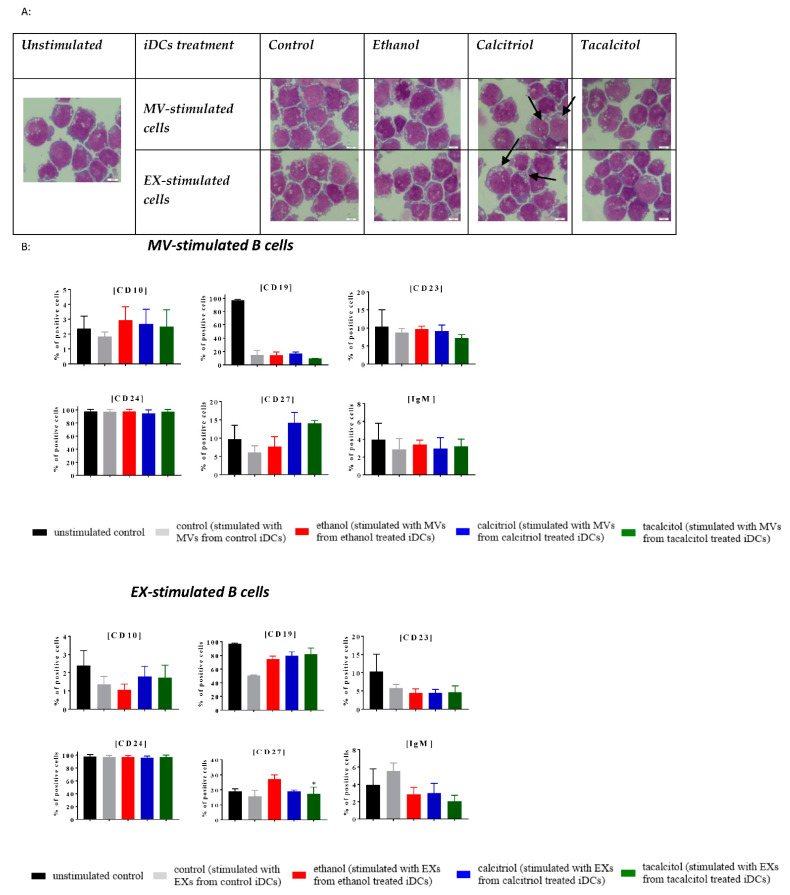
The indirect effects of calcitriol and tacalcitol on RCH-ACV cell line after 24 h stimulation with EVs derived from iDCs: (**A**) morphology of EV-stimulated RCH-ACV cell line; scale bar 10 μm. Each staining was performed twice; Black arrows indicate vacuolization in cells. (**B**) distribution of differentiation markers by flow cytometry analysis. Mean values and standard deviation are presented. Statistical analysis: Sidak’s multiple comparison tests in comparison with ethanol were performed; significant differences as compared with ethanol (* *p* < 0.005). Each experiment was performed at least 3 times.

**Figure 11:**
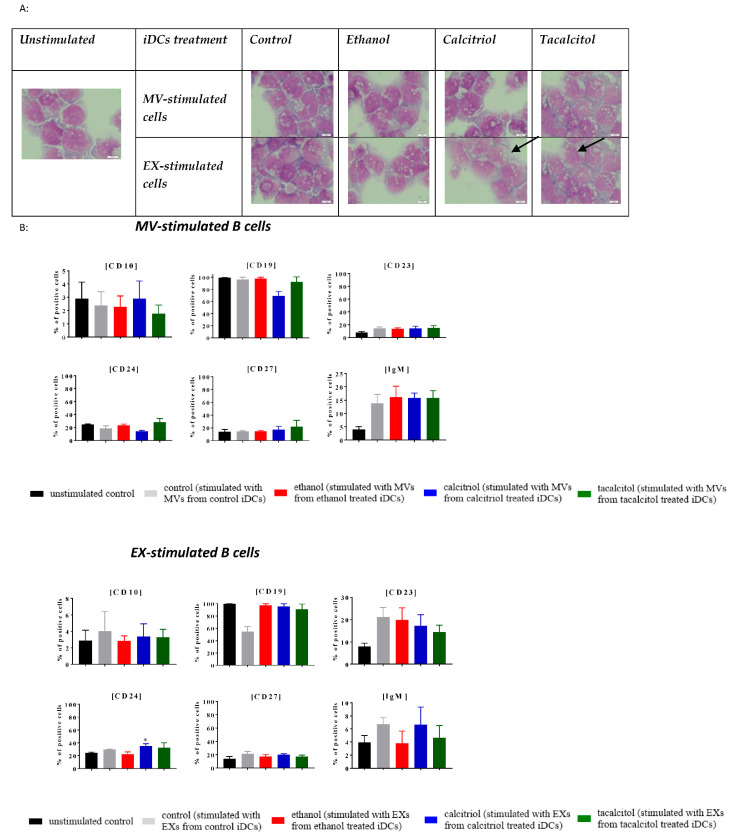
The indirect effect of calcitriol and tacalcitol on SEM cell line after 24 h stimulation with EVs derived from iDCs: (**A**) morphology of EV-stimulated SEM cells, scale bar 10 μm. Each staining was performed twice; Black arrows indicate vacuolization in cells. (**B**) distribution of differentiation markers by flow cytometry analysis. Mean values and standard deviation are presented. Statistical analysis: Sidak’s multiple comparison tests in comparison with ethanol were performed; significant differences as compared with ethanol (* *p* < 0.005). Each experiment was performed at least 3 times.

**Figure 12:**
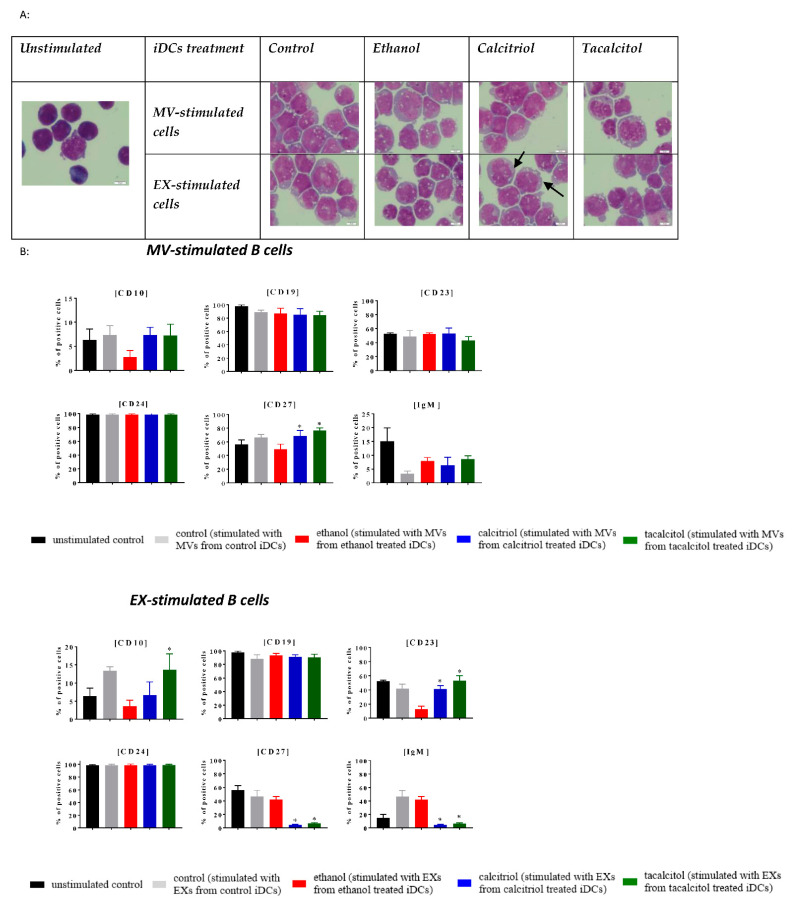
The indirect effects of calcitriol and tacalcitol on KOPN-8 cell line after 24 h stimulation with EVs derived from iDCs: (**A**) morphology of EV-stimulated KOPN-8 cells; scale bar 10 μm. Each staining was performed twice; Black arrows indicate vacuolization in cells. (**B**) distribution of differentiation markers by flow cytometry analysis. Mean values and standard deviation are presented. Statistical analysis: Sidak’s multiple comparison tests in comparison with ethanol were performed; significant differences as compared with ethanol (* *p* < 0.005). Each experiment was performed at least 3 times.

**Figure 13:**
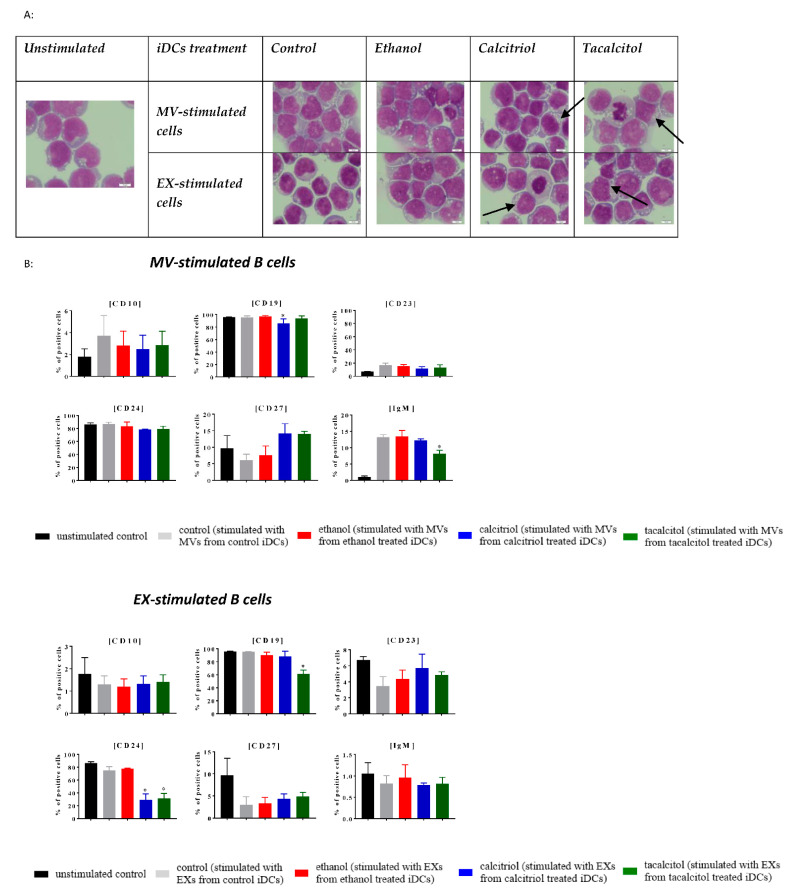
The indirect effects of calcitriol and tacalcitol on RS4;11 cell line after 24 h stimulation with EVs derived from iDCs: (**A**) morphology of EV-stimulated RS4,11 cell line; scale bar 10 μm. Each staining was performed twice; Black arrows indicate vacuolization in cells. (**B**) distribution of differentiation markers by flow cytometry analysis. Mean values and standard deviation are presented. Statistical analysis: Sidak’s multiple comparison tests in comparison with ethanol were performed; significant differences as compared with ethanol (* *p* < 0.005). Each experiment was performed at least 3 times.

Next, we compared the impact of EVs derived from iDCs on normal and leukemic B cells (Figure 9, Figure 10, Figure 11, Figure 12 and Figure 13).

We performed stimulation of normal (LCL) and leukemic B cells with the addition of EVs associated with the time of MP turnover in cells.

In all cell lines, we observed that EVs seemed to induce more vacuolization in the cytoplasm, compared with the unstimulated samples (Figure 9A, Figure 10A, Figure 11A, Figure 12A and Figure 13A; the effect in the cells is underlined by black arrows). In the RS4,11 cell line treated with MPs derived from VDA-stimulated iDCs, we observed an increased number of mitotic nuclei (Figure 13A).

Finally, we observed different effects on the phenotype of B cells, which, in turn, depended on the origin of MPs (MVs or EXs). The changes involved only a few CD markers: CD10, CD19, CD23, CD24, CD27, and IgM (Figure 9B, Figure 10B, Figure 11B, Figure 12B and Figure 13B). The results illustrating other phenotype markers analyzed are presented in Supplementary Files (Figures S8–S12).

Incubation of control and leukemic cell lines with MVs derived from iDCs treated with calcitriol and/or tacalcitol showed the following changes in the expression of the above-mentioned markers:

➢ LCL cell line: increase in CD10, IgM, and potentially significantly in CD23 (Figure 9B);
➢ RCH-ACV and SEM cell lines: no significant phenotypic changes (Figure 10B and Figure 11B);
➢ KOPN-8 cell line: increase in CD27 (Figure 12B);
➢ RS4;11 cell line: decrease in CD19 and IgM (Figure 13B).

In other situations, we observed the following results regarding samples cultured in the presence of EXs derived from myeloid iDCs stimulated with calcitriol and/or tacalcitol:

➢ LCL cell line: decrease in CD19 (Figure 9B);
➢ RCH-ACV cell line: decrease in CD27 (Figure 10B);
➢ SEM cell line: increase in CD24 (Figure 11B);
➢ KOPN-8 cell line: decrease in CD27 and IgM and increase in CD10 and CD23 (Figure 12B);
➢ RS4;11 cell line: decrease in CD19 and CD24 (Figure 13B).

Additionally, in our study, we sought to assess if MVs and EXs secreted by iDCs stimulated with VDAs could impact the release of IgM or IgG by precursor B cells. We measured total IgG and IgM concentration in unstimulated versus stimulated cells with EV samples (Supplementary Files Figure S13).

Unstimulated cells did not release IgG or IgM, with the exception of tacalcitol-treated RS4;11 and RCH-ACV cell lines (Supplementary Files Figure S13).

## 4. Discussion

According to the previous studies on ALL-B cells, calcitriol inhibits the growth of malignant B cells without the induction of a cytotoxic effect. A similar effect (inhibition of proliferation and immunoglobulin secretion) of calcitriol was observed in normal B cells [33,34,35]. DCs are important in B cell proliferation and survival, and calcitriol also affects their function [36]. Therefore, in our studies, we compared the direct effects exerted by calcitriol on normal B cell and ALL-B cell lines with the effects evoked by EVs derived from iDCs incubated with calcitriol and tacalcitol.

For this purpose, we selected the SC cell line, the monocyte/macrophage cell line for which we did not find any information in the literature regarding phenotype changes after incubation with VDAs; however, according to our previous studies [29], as well as from studies of the other teams, e.g., [37,38,39], we supposed that such type of cell line could be sensitive to VDAs. We confirmed the sensitivity of parental and iDCs on VDAs by checking the cell cycle distribution and discrimination between early and late apoptotic and necrosis. Next, we noticed that VDAs could increase the vacuolization process and could also increase the percentage of CD markers associated with the maturation of the cells, which encouraged us to determine the impact of VDAs on the release of the most known types of EV, so we characterized the phenotype changes in the MVs and EXs released from our generated cells, and the impact of VDAs on their size, morphology, and their ability to modulate the CD marker expression on normal and leukemic B cells. It was found that calcitriol and tacalcitol changed the phenotype of iDCs, as well as EVs derived from them. Moreover, calcitriol and tacalcitol affected the phenotype of EVs derived from monocyte cell line SC (parental for iDCs). To date, few data in the literature describe the impact of calcitriol on endothelial-cell-derived [40] and osteoclast-derived EVs [41]. Bruns et al. described vitamin D-mediated inhibition of CLL-cell-mediated myeloid-derived suppressor cells (MDSC) induction by exosomal transfer of miR-155 [42]. However, the influence of calcitriol or its analogs on DC-derived EVs was not reported so far. Despite significant phenotypic changes caused by calcitriol and tacalcitol on DCs (significant increases in CD11c, CD14, and CD16 by both compounds), the characteristics of MVs were influenced only by tacalcitol, which showed decreased CD11c. A greater impact of calcitriol was observed on EXs derived from DCs, significantly increasing the levels of CD9 and CD63. Tacalcitol increased the percentage of CD81-positive EXs. In addition, calcitriol increased the size of EXs. Both vitamin D analogs increased the percentage of CD11b- and CD86-positive EXs, which could indicate the role of EXs in the immune response regulation. Normal B cells and ALL cells were treated with EVs characterized in this way.

In our studies, we did not observe any significant inhibitory effect of calcitriol or tacalcitol on normal LCL B cell line, ALL cells with MLL translocation, or ALL cells with other mutations. The exception is 20–30% proliferation inhibition of the RCH-ACV cell line (without MLL translocation) by both compounds at 100 nM concentrations. A similar proliferation inhibition rate was observed by Kozielewicz et al. in their studies on diffuse large B cell lymphoma (DLBCL) cell lines with the use of calcitriol and tacalcitol [43].

Among the analyzed surface molecules, only a few of them showed differences in expression between ethanol-treated control cells and cells treated with VDAs or EVs. CD19 is a molecule that showed diminished expression regardless of whether cells were treated directly with both VDAs or with EVs. This effect was also independent of the type of cell line used for experiments. A similar effect of calcitriol was also noticed on peripheral blood mononuclear cells (PBMCs) derived from patients with systemic lupus erythematosus (SLE), who showed a decrease in the constitutive autoantibody production [44]. The expression of CD19 appears at the point of B lineage incitation and continues during mature B cell differentiation. Finally, the expression is downregulated during terminal differentiation into plasma cells [45]. Contrary to these results are the findings of our other studies in mice bearing 4T1 mouse mammary gland tumors and treated with calcitriol or its analogs, in which we observed increased blood levels of CD19-positive cells. These differences may result from the tumor milieu influencing the whole-body response to the treatment [46]. In an interesting review, Rolf et al. discussed the differences that were observed between the in vitro and in vivo studies with the use of calcitriol or VDAs, emphasizing the greater impact of a germinal center and survival niches [47].

CD27 was another antigen with a decreased expression on RCH-ACV and KOPN-8 cell lines by EXs derived from iDCs stimulated with calcitriol and tacalcitol. The results were in agreement with those found in the studies by Haas et al. conducted on patients with multiple sclerosis (MS), which showed that hypovitaminosis D enhances the accumulation of antigen-experienced CD27-positive cells expressing isotype-switched antibodies, as well as mature plasma cells [48]. On the other hand, CD27 expression increased on LCL and RCH-ACV cell lines by direct treatment with calcitriol and tacalcitol and on KOPN-8 cell line incubated with MVs derived from DCs treated with VDAs. The expression of CD27 decides the response to the antigen stimulation (the presence of CD27 marker is associated with the release of 5–100-fold more Igs) and the B cells expressing CD27 on their surface are identified as human memory B cells [49]. Moreover, high *CD27* levels represent a poor prognostic marker for high-risk pediatric pro-B ALL cells [50].

Both compounds decreased the percentage of CD24-positive cells in the SEM cell line, but when the SEM cell line was incubated with EXs from DCs treated with calcitriol, the expression of CD24 increased. EXs (DCs derived after treatment with calcitriol and tacalcitol) decreased CD24 expression in RS4;11 cell line. This marker mostly serves as a costimulatory factor of T cells, is involved in B cell activation and differentiation, and could be important to distinguish pre-pro-B, and pro-B cells from pre-B cells [51]. Decreased expression of this antigen on leukemic cells may be unfavorable, because of the results of studies showing that B-lineage ALL from children lacking the expression of CD24 is associated with radiation resistance [50]. Additionally, the expression of the percentage of CD38 in calcitriol- and tacalcitol-treated SEM cell lines was significantly increased. According to the literature, it is known that CD38 is a type of II transmembrane glycoprotein that, in humans, acts as a signaling channel that leads to the activation and proliferation of cells [52]. Interestingly, in our studies, calcitriol, and to a lesser extent tacalcitol (10 nM or 1 nM), stimulated the proliferation of SEM cell line in short (24 h) culture, which highlighted the possibility of unfavorable effects of calcitriol supplementation in this type of ALL.

Although CD10 is called a common acute lymphoblastic leukemia antigen (CALLA), its expression on cell lines tested was surprisingly low. The expression of CD10 is transient during B cell maturation until the pre-B cell stage [53]. Therefore, the observed increase in CD10 expression by EVs derived from DCs treated with vitamin D compounds in LCL (MVs) and KOPN-8 (EXs) cell lines may reflect the impact of calcitriol on B cell differentiation—namely, the inhibition of generation of plasma cells and memory B cells [35]. The same pattern of expression as that by EXs derived from iDCs on the KOPN-8 cell line and potentially by MVs derived from iDCs on the LCL cell line was observed for the CD23 molecule. Physiologically, CD23 is a low-affinity IgE receptor. Transitional B cells expressing CD23 proliferate faster and are less susceptible to BCR-induced apoptosis [54]. Moreover, the loss of CD23 expression is described as a consequence of B cell activation [55]. The inhibition of B cell activation by calcitriol was previously described by Hartmann et al. in 2011. Moreover, in the case of hematological malignancies, the CD23-negative CD19+CD5+ cells are related to worse prognoses [56,57].

We also observed increased expression of IgM in RS4;11 cell lines with calcitriol treatments and in normal LCL cell lines after incubation with MVs. An opposite effect was observed in the RS4;11 cell line after incubation with MVs and in the KOPN-8 cell line incubated with EXs derived from VDA-treated DCs. On the other hand, our experiments showed no impact of calcitriol and tacalcitol (direct or through impact on EVs) in IgG released by B cells. The terminal stage of B cell differentiation associated with antibody secretion is represented by plasma cells. Calcitriol suppresses the production of Th1 cytokines but enhances Th2 once it becomes involved in humoral immune response promotion [58]. According to studies by Rolf et al., calcitriol inhibits the antibody secretion in in vitro conditions by human PBMCs stimulated with either PWM (pokeweed mitogen) or dermatophyte O. Such effect is possible through other immune cells or by a direct effect on B cells. Moreover, Chen et al. demonstrated that calcitriol decreases the percentage of the absolute number of plasma cells, together with a decrease in the secretion of IgA, IgE, IgG, and IgM [35,59].

Actually, there is a lack of optimal therapy regimens for patients with *MLL* translocations. Cytotoxic and cytoreductive chemotherapy is still the standard protocol, which includes a steroid (dexamethasone or prednisolone) in combination with vincristine, and in some patients, also asparaginase and anthracyclines. Toxicity and long-term complications limit the usefulness of standard chemotherapy, especially in young patients [60,61,62,63]. These observations implicate that further studies are needed to evaluate new approaches to ALL-B leukemia treatment in vitro and in vivo. The significance of VDAs, in this case, is supported by the effect of calcitriol and its analog (direct or through EVs) on the CD expression pattern, which correlates with the prognosis and effectiveness of treatment in ALL-B. The high expressions of CD27 (considered a marker of poor prognosis) and CD24 (related to low response to radiotherapy) are associated with poor prognosis, while high expressions of CD38 [64] and CD23 [58] are recognized as good prognostic factors.

Therefore, for the SEM cell line, it seems that the direct effect of calcitriol should be favorable, decreasing CD24 and increasing CD38 expression, but this effect could be modified in the organism by the effect of calcitriol on, for example, DCs and its impact on SEM cell line (increased expression of CD24 by EXs from calcitriol-treated DCs). Interesting results were observed on the KOPN-8 cell line, in which a direct effect of VDAs was limited only to decreased CD19 expression, while MVs and EXs derived from calcitriol and tacalcitol acted in an opposite manner on the expression of CD27. Therefore, the final effect observed in vivo was counterintuitive.

The comparison of the direct and indirect impacts of calcitriol and tacalcitol on the phenotype of normal and leukemic B cells is summarized below (Table 2).

The functional immune system could destroy leukemic cells, so studies based on their cooperation are required. Our observation that stimulation of myeloid iDCs by calcitriol and tacalcitol could influence the phenotype of leukemic B cells by releasing EVs suggests the ability of vitamin D and its analogs to regulate anti-leukemic humoral response by direct interaction with VDR and cytokines/chemokines or growth factors, but also by cellular communication through EVs. Therefore, further studies to explain the modulation of the composition of EVs by vitamin D compounds are required.

## Figures and Tables

**Table 1:** Monoclonal antibodies used in the studies.

Cells/Microvesicles/Exosomes	Antibody (Clone) Fluorescent Probe	Microvesicles/Exosomes	Antibody (Clone) Fluorescent Probe
**Monocyte**	CD9 (M-L13) PE-CF594	**Exosomes (monocyte)**	CD9 (M-L13) PE-CF594
	CD11b PE		CD81 (JS-81) APC
	CD14 (M5E2) APC		CD63(H5C6) PE
	CD16 (3G8) BV421		HLA-DR (G46-6) BV421
	HLA-DR (G46-6) BV421	**Exosomes (dendritic cell)**	CD9 (M-L13) PE-CF594
**Dendritic cell (immature/mature)**	CD9(M-L13) PE-CF594		CD81 (JS-81) APC
	CD11c (B-ly6) APC		CD63 (H5C6) PE
	CD11b PE		HLA-DR (G46-6) BV421
	CD81 (JS-81) APC		CD11b PE
	CD123 (7G3) PE		CD40 (5C3) PE
	CD14 (M5E2) APC		CD86 (FUN-1)APC
	CD16 (3G8) BV421		Annexin V APC
	HLA-DR (G46-6) BV421	**Microvesicles (monocyte)**	CD11b PE
**Precursor B cell (before/after stimulation by microvesicles/exosomes) (monocyte)**	CD10 (HI10a) BV421		CD14(M5E2) APC
	CD19 (HIB19) PE		HLA-DR (G46-6)BV421
	CD20 (2H7) APC	**Microvesicles (dendritic cell)**	CD11c (B-ly6) APC
	CD23 (M-L233) BB700		CD123(7G3) PE
	CD24 (ML5) BV421		CD11b PE
	CD27 (M-T271) BV421		CD14 (M5E2) APC
	CD34 APC		HLA-DR(G46-6) BV421
	CD38 (HIT2) BB515		
	HLA-DR(G46-6) BV421		
	IgM (G20-127) APC		
	IgD (IA6-2) PE		

To exclude dead cells, (PI) staining was performed. Each experiment was repeated at least 3 times.

**Table 2:** The direct/indirect impacts of calcitriol/tacalcitol on normal and leukemic B cells.

		LCL	RCH-ACV	SEM	KOPN-8	RS4;11
Translocation		-	t(11,19)	t(4;11)	t(4;11)	t(4;11)
Calcitriol/Tacalcitol cell stimulation						
Direct		CD19↓ CD27↑↑	CD19↓↓ CD27↑	CD24↓↓ CD38↑↑	CD19↓	IgM↑
Indirect	MVs	CD10↑ CD23↑↑ * IgM↑	-	-	CD27↑↑	CD19↓ IgM↓
	EXs	CD19↓	CD27↓	CD24↑	CD10↑ CD23↑↑ CD27↓↓ IgM↓↓	CD19↓ CD24↓↓

Potential statistical significance (the difference between MVs derived from iDCs stimulated with ethanol and MVs derived from iDCs stimulated with VDAs) was less than twofold. In order to make the table more transparent, different colors were assigned to each marker. Red color was assigned with the expression of CD19, dark blue with CD27, green with CD24, orange with CD38, violet with IgM, black with CD10 and grey with CD23. The arrows ↑ indicates the increase of the expression, and the ↓ indicates the decrease. The strong of the expression was visualized by the two arrows. Abbreviations: (*) means potentially statistically significant.

## Data Availability

The data used and analyzed during the current studies are available from the corresponding author upon reasonable request.

## Supplementary Materials

The following are available online at https://www.mdpi.com/article/10.3390/jcm11082224/s1. Figure S1: Analysis of apoptosis using Annexin V and PI staining upon calcitriol and tacalcitol stimulation, Figure S2: Cell cycle distribution upon calcitriol and tacalcitol stimulation, Figure S3: Kinetics of anti-proliferative effects of calcitriol and tacalcitol against normal B cell line (LCL) after 48 h, 72 h, 120 h, 144 h, and 168 h, Figure S4: Kinetics of anti-proliferative effects of calcitriol and tacalcitol against leukemic B cell line (RCH-ACV) after 48 h, 72 h, 120 h, 144 h, and 168 h, Figure S5: Kinetics of anti-proliferative effects of calcitriol and tacalcitol against leukemic B cell line (SEM) after 48 h, 72 h, 120 h, 144 h, and 168 h, Figure S6: Kinetics of anti-proliferative effects of calcitriol and tacalcitol against leukemic B cell line (KOPN-8) after 48 h, 72 h, 120 h, 144 h, and 168 h, Figure S7: Kinetics of anti-proliferative effects of calcitriol and tacalcitol against leukemic B cell line (RS4;11) after 48 h, 72 h, 120 h, 144 h, and 168 h, Figure S8: The indirect effect of calcitriol and tacalcitol on LCL cell line after 24 h of stimulation with myeloid-dendritic-cell-derived EVs (distribution of differentiation markers by flow cytometry analysis), Figure S9: The indirect effect of calcitriol and tacalcitol on RCH-ACV cell line after 24 h of stimulation with myeloid-dendritic-cell-derived EVs (distribution of differentiation markers by flow cytometry analysis), Figure S10: The indirect effect of calcitriol and tacalcitol on SEM cell line after 24 h of stimulation with myeloid-dendritic-cell-derived EVs (distribution of differentiation markers by flow cytometry analysis), Figure S11: The indirect effect of calcitriol and tacalcitol on KOPN-8 cell line after 24 h of stimulation with myeloid-dendritic-cell-derived EVs (distribution of differentiation markers by flow cytometry analysis), Figure S12: The indirect effect of calcitriol and tacalcitol on RS4;11 cell line after 24 h stimulation with myeloid dendritic-cell-derived EVs (distribution of differentiation markers by flow cytometry analysis), Figure S13: Total IgG concentration (g/l) after 24 h of stimulation with myeloid dendritic cells MVs/EXs, Table S1: The direct effect of calcitriol and tacalcitol on the distribution of CD markers on normal and leukemic B cells. Flow cytometry distribution of CD markers.

## Abbreviations

**VDA**	Vitamin D analog
**ALL**	Acute lymphoblastic leukemia
**VDR**	Vitamin D receptor
**MV**	Microvesicle
**EX**	Exosome
**mDCs**	Myeloid dendritic cells
**iDCs**	Immature dendritic cells
**matDCs**	Mature dendritic cells
**EV**	Extracellular vesicle
**APCs**	Antigen-presenting cells
**pDCs**	Plasmacytoid dendritic cells
**MHC**	Major histocompatibility complex
**GM-CSF**	Colony-stimulating granulocyte-macrophage factor
**IL**	Interleukin
**ATRA**	All-trans retinoic acid
**PMA**	Phorbol 12-myristate 13-acetate
**1,25 (OH)_2_D_3_**	Calcitriol
**Treg**	Regulatory T cell
**MP**	Microparticle
**MVBs**	Multivesicular bodies
**ILVs**	Intraluminal vesicles
**SNARE**	SNAP receptor
**Tsg 101**	Tumor susceptibility gene 101
***MLL***	Mixed-lineage leukemia
**DFS**	Disease-free survival
**AF**	Acute lymphoblastic leukemia 1-fused gene
**AML**	Acute myeloid leukemia
**CYP27B1**	25-Hydroxyvitamin D3-1a-hydroxylase
**Th**	Helper T cell
**CDKN**	Vyclin-dependent kinase inhibitor
**ATCC**	American Type Culture Collection
**DSMZ**	German Collection of Microorganisms and Cell Culture
**EBV**	Epstein–Barr virus
**IMDM**	Iscove modified Dulbecco medium
**FBS**	Fetal bovine serum
**BSA**	Bovine serum albumin
**RPMI-1640**	Medium Roswell Park Memorial Institute 1640
**MEMα**	Minimal essential medium
**1,24*R* (OH)_2_D_3_/ PRI-2191**	Tacalcitol
**MTT**	Colorimetric assay for assessing cell metabolic activity
**DMSO**	Dimethyl sulfoxide
**IC_50_**	Half-maximal inhibitory concentration
**MGG**	May–Grunwald–Giemsa staining
**PI**	Propidium iodide
**BSA**	Bovine serum albumin
**FACS**	Fluorescence-activated cell sorter
**DLS**	Dynamic light scattering
**Cryo-LV-FESEM**	Cryogenic low-voltage field-emission scanning Electron microscopy
**ET**	Everhart–Thornley detector
**SD**	Standard deviation
**PdI**	Polydispersity index
**CLL**	Chronic lymphoblastic leukemia
**MDSC**	Myeloid-derived suppressor cell
**DLBCL**	Diffuse large B-cell lymphoma
**PBMC**	Peripheral blood mononuclear cell
**SLE**	Systemic lupus erythematosus
**MS**	Multiple sclerosis
**IgV**	Immunoglobulin variable domain
**CALLA**	Common acute lymphoblastic leukemia antigen
**BCR**	B cell receptor
**PWM**	Pokeweed mitogen
