# Supplementary material for: The Impact of Exosomes/Microvesicles Derived from Myeloid Dendritic Cells Cultured in the Presence of Calcitriol and Tacalcitol on Acute B-Cell Precursor Cell Lines with MLL Fusion Gene
Source: J Clin Med. 2022 Apr 15;11(8):2224. doi: 10.3390/jcm11082224 (PMC9032710; doi:10.3390/jcm11082224)
Supplement: Supplementary file 1 [file jcm-11-02224-s001.zip › jcm-1640773-supplementary.pdf]

Supplementary Files:

The Impact of Exosomes/Microvesicles Derived from Myeloid Dendritic Cells Cultured in the Presence of Calcitriol and Tacalcitol on Acute B-cell Precursor Cell Lines with *MLL* Fusion Gene

Eliza Turlej[1], Tomasz M. Goszczyński[2], Marek Drab[3], Beata Orzechowska[4], Magdalena Maciejewska[5], Joanna Banach[5], Joanna Wietrzyk[5]

[1] Department of Experimental Biology, Wroclaw University of Environmental and Life Sciences, 50-375 Wrocław, Poland

[2] Laboratory of Biomedical Chemistry, Hirszfeld Institute of Immunology and Experimental Therapy, Polish Academy of Sciences, 53-114 Wroclaw, Poland

[3] Laboratory of Interactions of Biological Nanostructures, Department of Immunology of Infectious Diseases, Hirszfeld Institute of Immunology and Experimental Therapy, Polish Academy of Sciences, 53-114 Wroclaw, Poland

[4] Laboratory of Virology, Department of Immunology of Infectious Diseases, Hirszfeld Institute of Immunology and Experimental Therapy, Polish Academy of Sciences, 53-114 Wroclaw, Poland

[5] Department of Experimental Oncology, Hirszfeld Institute of Immunology and Experimental Therapy, Polish Academy of Sciences, 53-114 Wroclaw, Poland

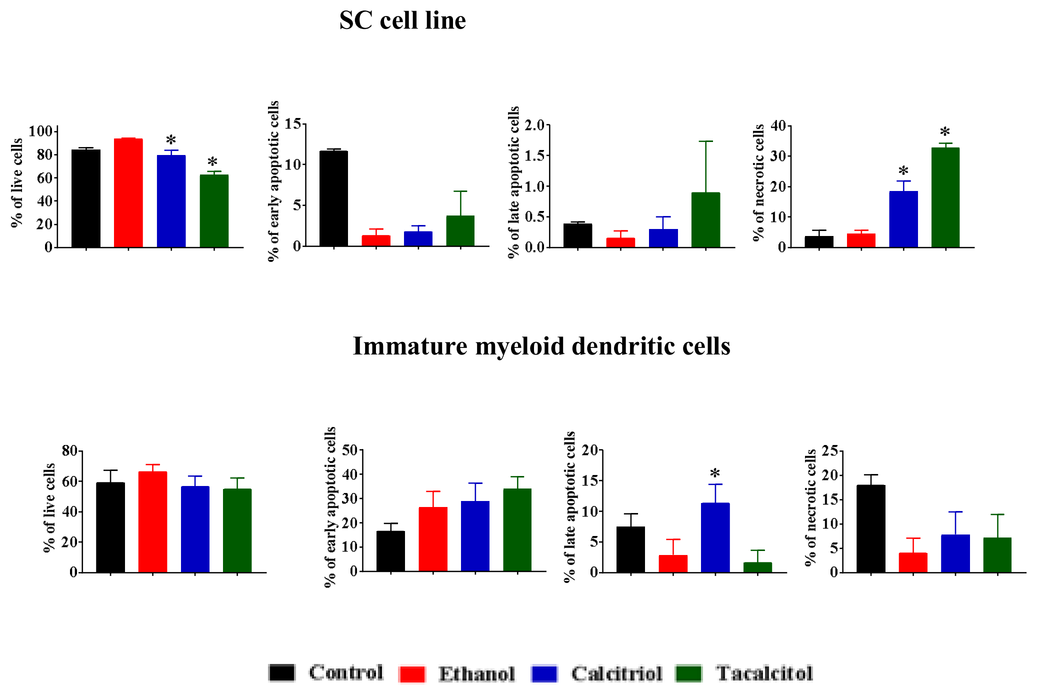

Figure S1. Analysis of apoptosis using Annexin V and PI staining upon calcitriol and tacalcitol stimulation.

Mean values and standard deviation are presented**.** Statistical analysis: Sidak's multiple comparisons test; significant differences as compared to ethanol (* $p < 0.005$). The graphs represent data from three separated experiments.

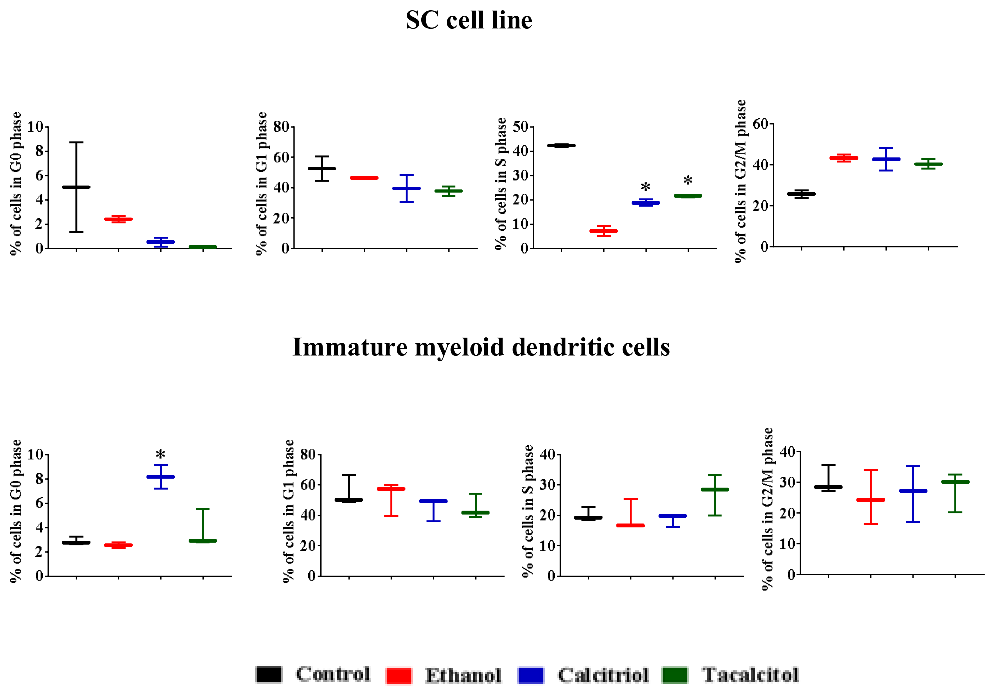

Figure S2. Cell cycle distribution upon calcitriol and tacalcitol stimulation.

Mean values and standard deviation are presented. Statistical analysis: Sidak's multiple comparisons test; significant differences as compared to ethanol (* $p < 0.005$). The graphs represent data from three separated experiments.

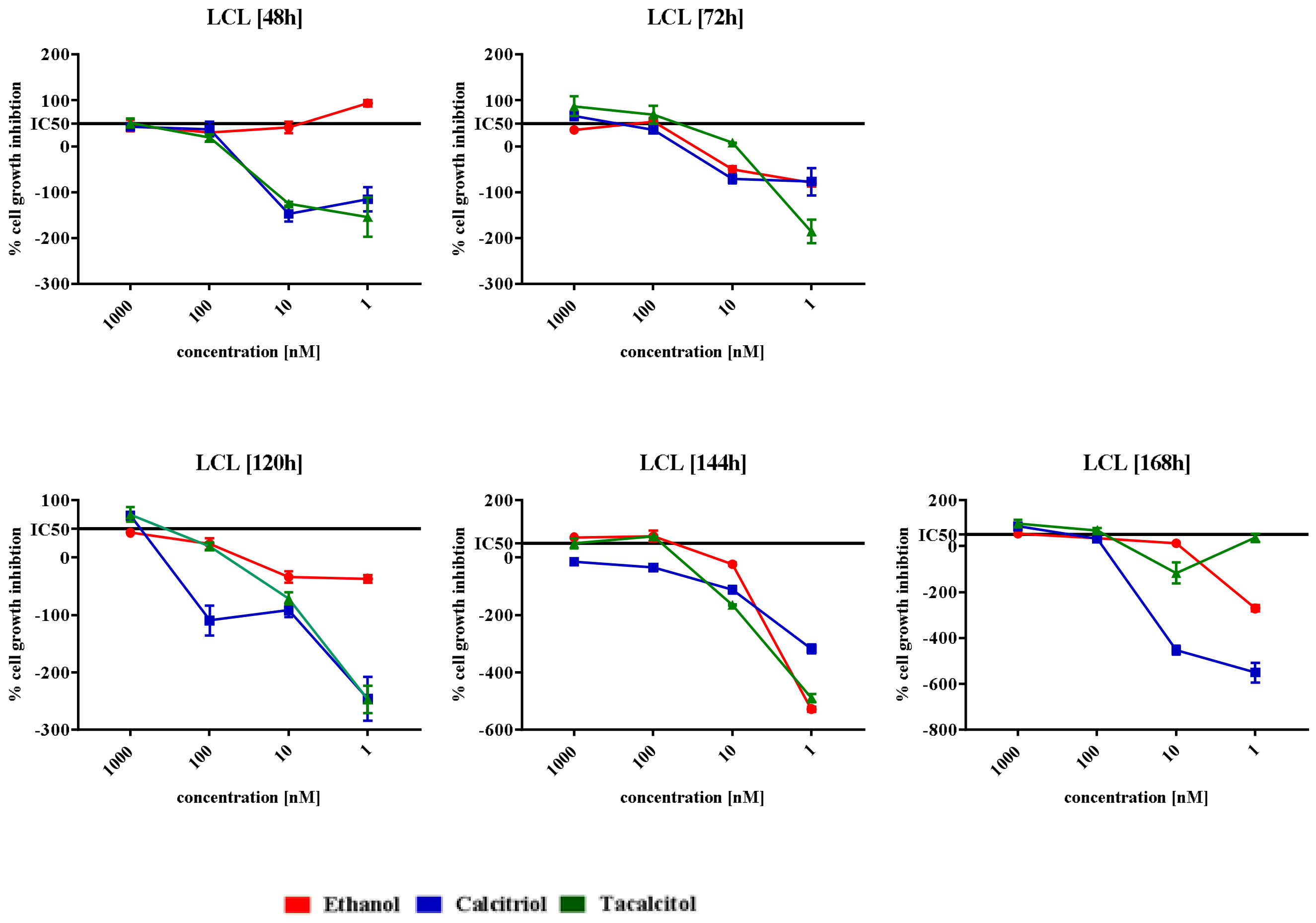

Figure S3. Kinetics of anti-proliferative effects of calcitriol and tacalcitol against normal B cell line (LCL) after 48 h, 72 h, 120 h, 144 h and 168 h.

Mean values and standard deviation are presented. Each measurement of anti-cytotoxicity effect (at each time point and at each concentration) was performed at least three times independently.

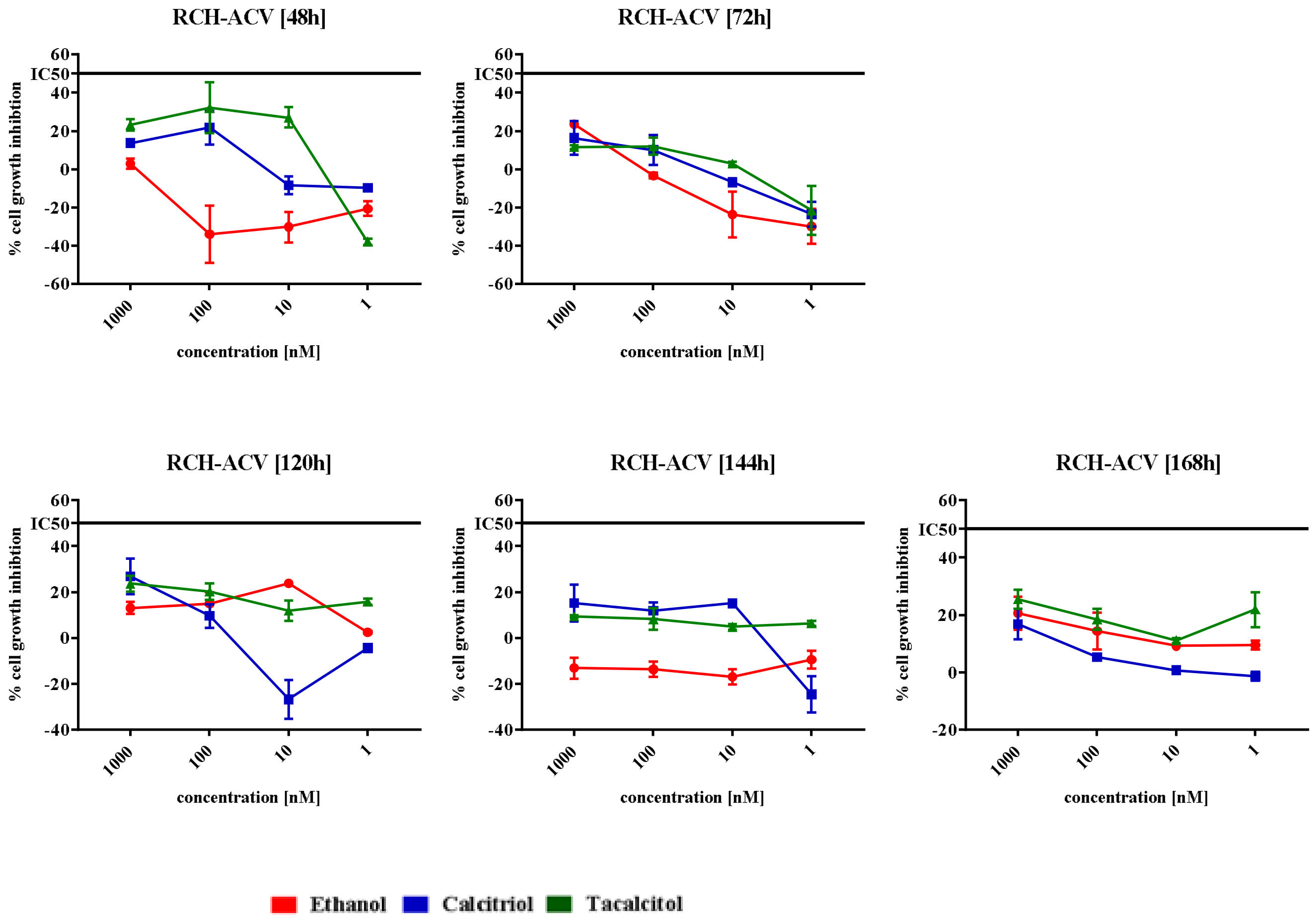

Figure S4. Kinetics of anti-proliferative effects of calcitriol and tacalcitol against leukemic B cell line (RCH-ACV) after 48 h, 72 h, 120 h, 144 h and 168 h.

Mean values and standard deviation are presented. Each measurement of anti-cytotoxicity effect (at each time point and at each concentration) was performed at least three times independently).

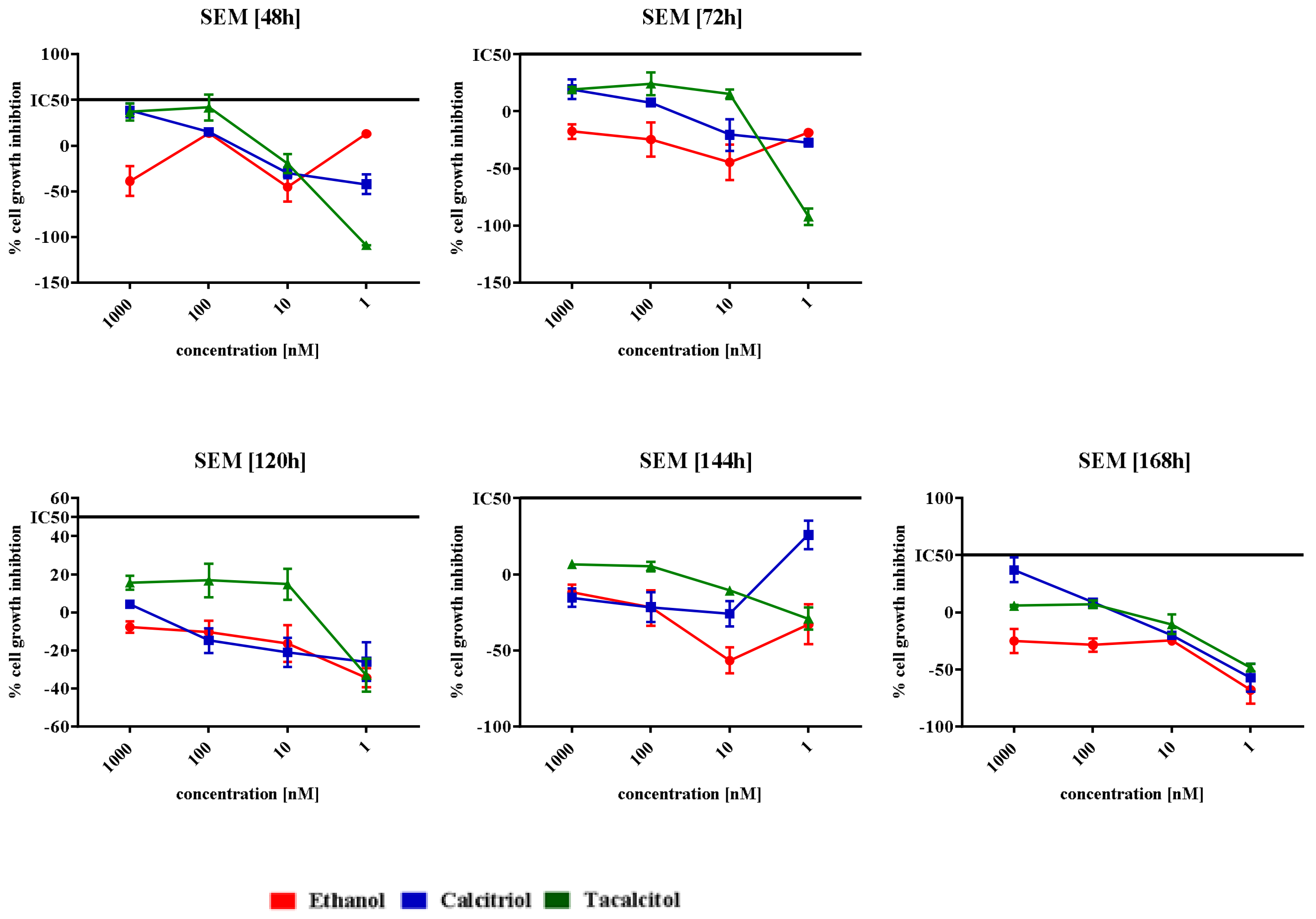

Figure S5. Kinetics of anti-proliferative effects of calcitriol and tacalcitol against leukemic B cell line (SEM) after 48 h, 72 h, 120 h, 144 h and 168 h.

Mean values and standard deviation are presented. Each measurement of anti-cytotoxicity effect (at each time point and at each concentration) was performed at least three times independently).

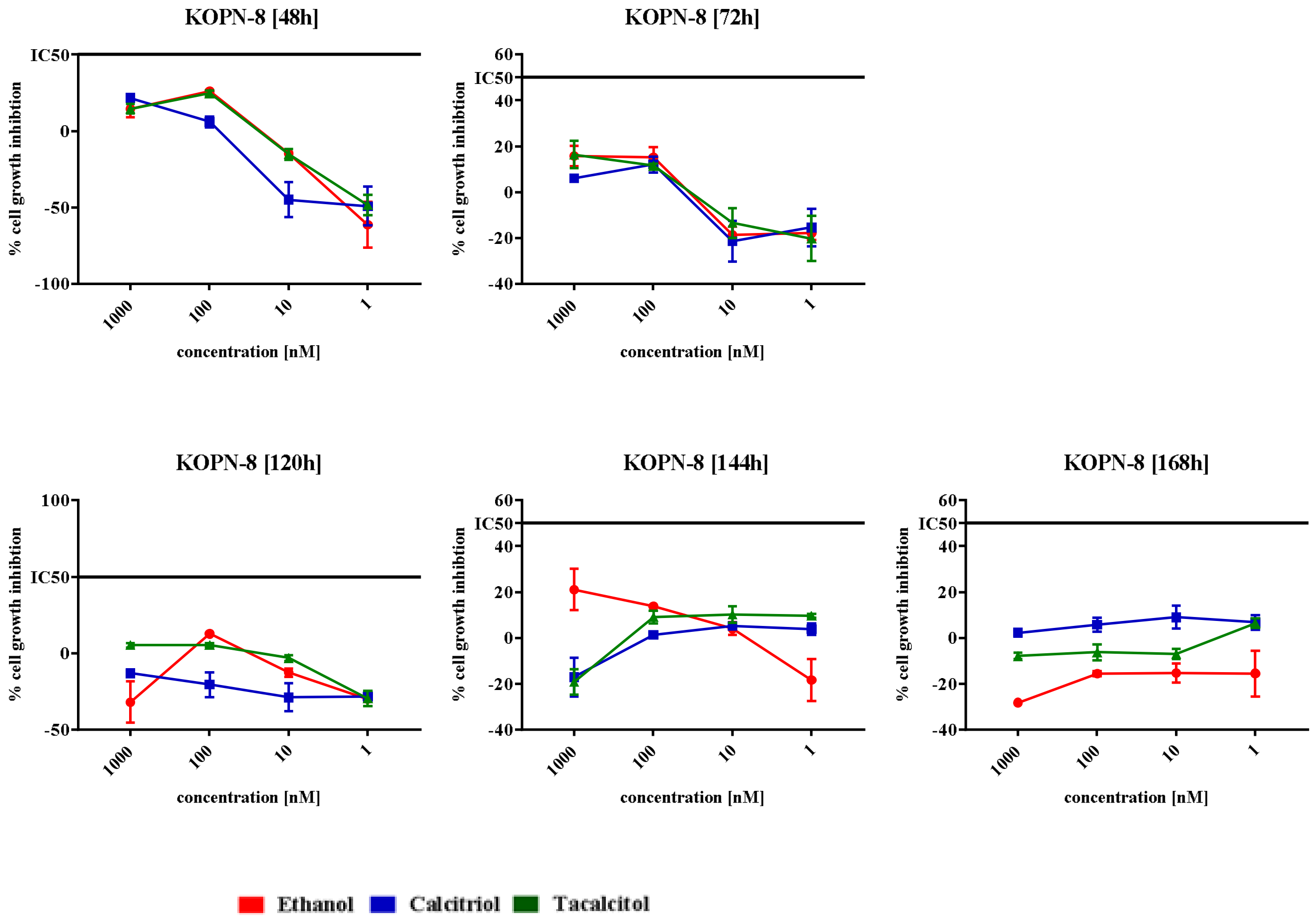

Figure S6. Kinetics of anti-proliferative effects of calcitriol and tacalcitol against leukemic B cell line (KOPN-8) after 48 h, 72 h, 120 h, 144 h and 168 h.

Mean values and standard deviation are presented. Each measurement of anti-cytotoxicity effect (at each time point and at each concentration) was performed at least three times independently).

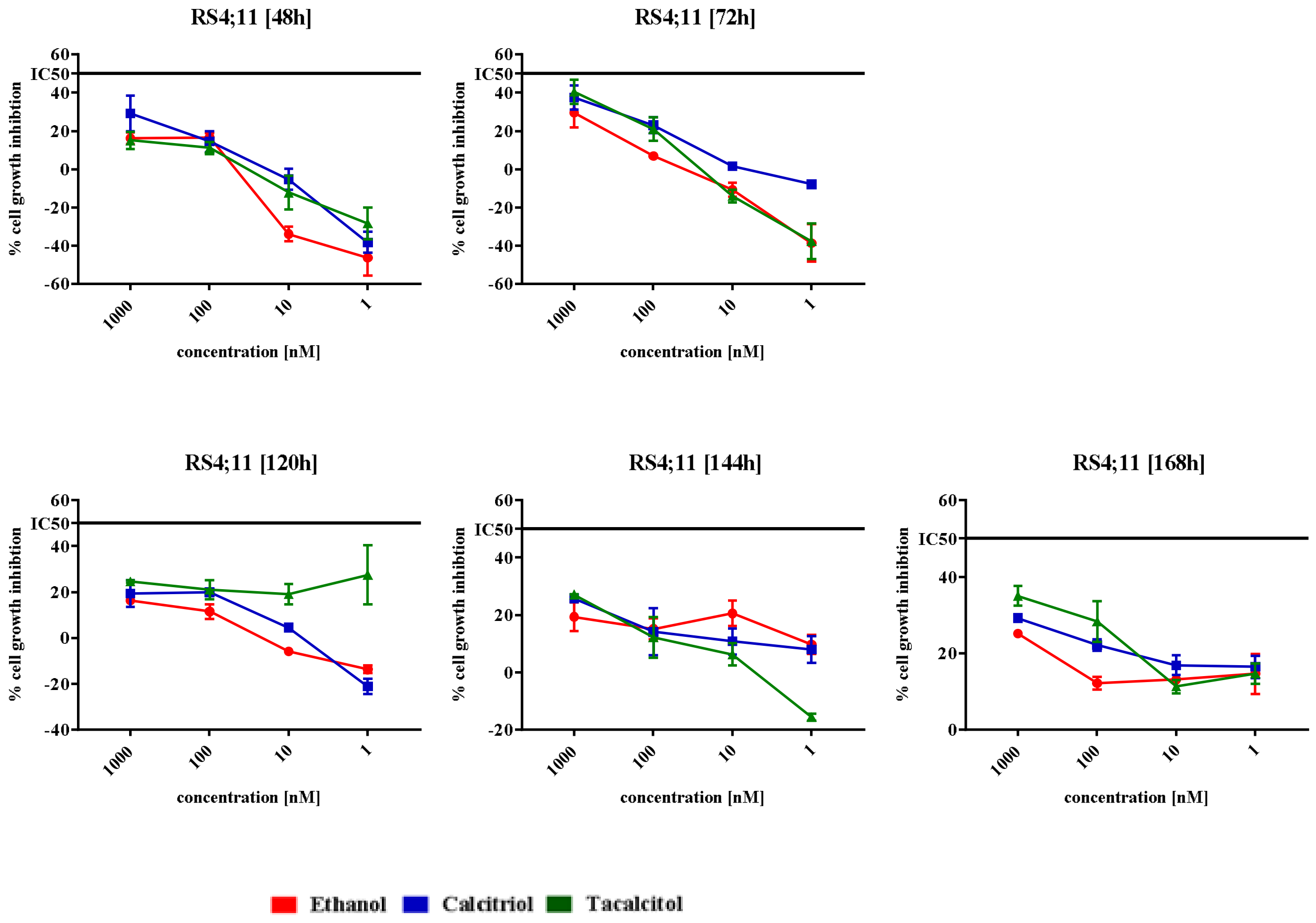

Figure S7. Kinetics of anti-proliferative effects of calcitriol and tacalcitol against leukemic B cell line (RS4;11) after 48 h, 72 h, 120 h, 144 h and 168 h.

Mean values and standard deviation are presented. Each measurement of anti-cytotoxicity effect (at each time point and at each concentration) was performed at least three times independently).

Microvesicles

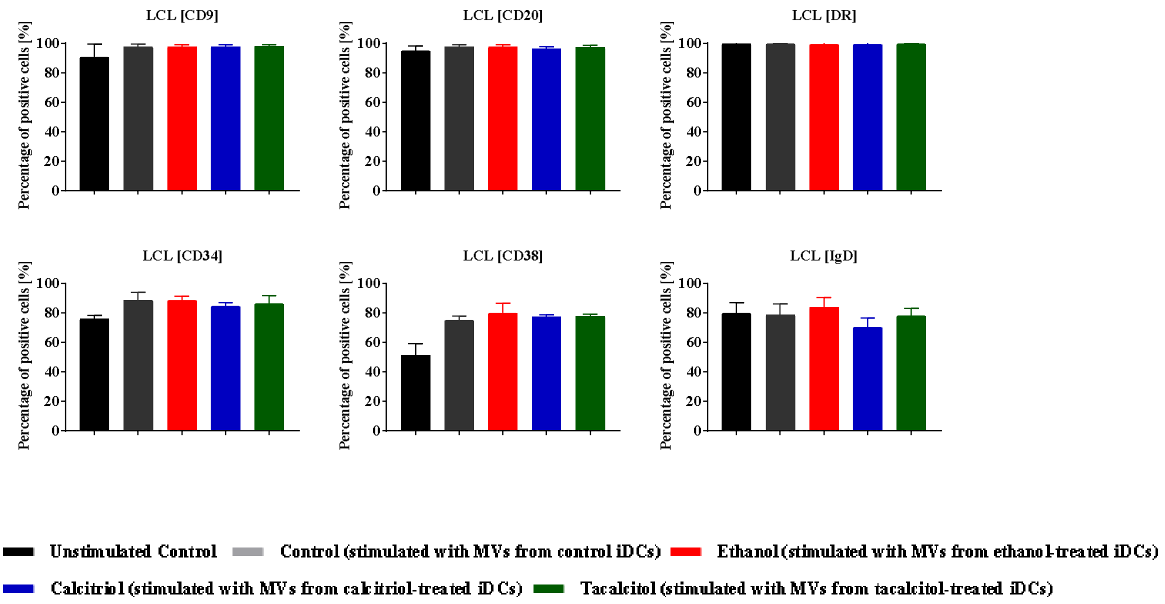

Exosomes

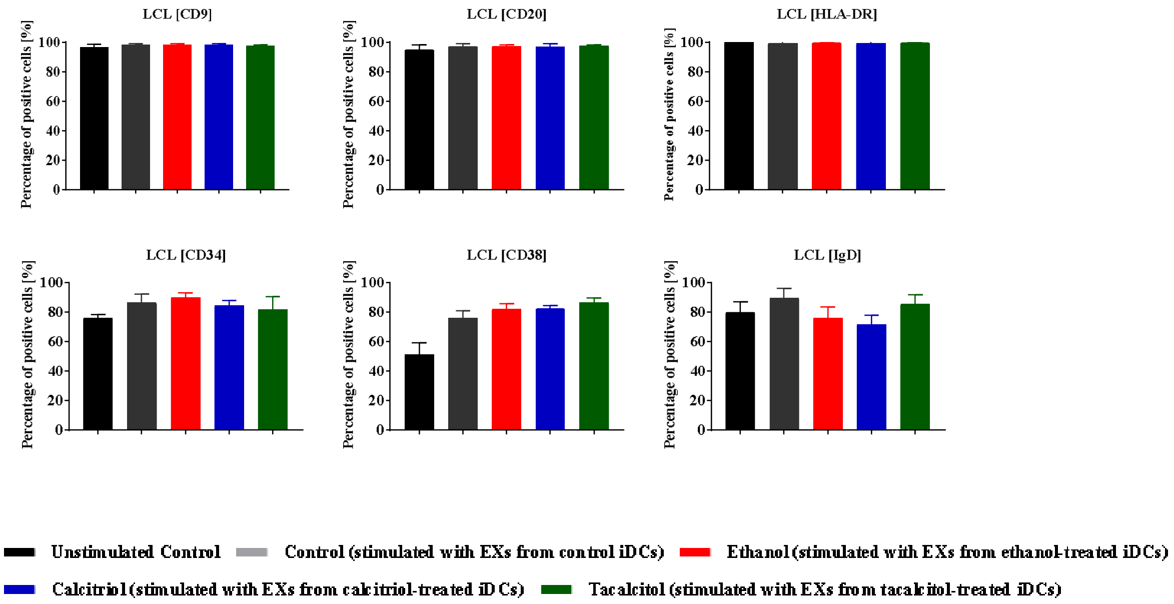

Figure S8. The indirect effect of calcitriol and tacalcitol on LCL cell line after 24 h of stimulation with myeloid dendritic cells - derived EVs (distribution of differentiation markers by flow cytometry analysis).

Mean values and standard deviation are presented. Statistical analysis: Sidak's multiple comparison in comparison to ethanol were performed. The graphs represent data from at least three separate experiments

Microvesicles

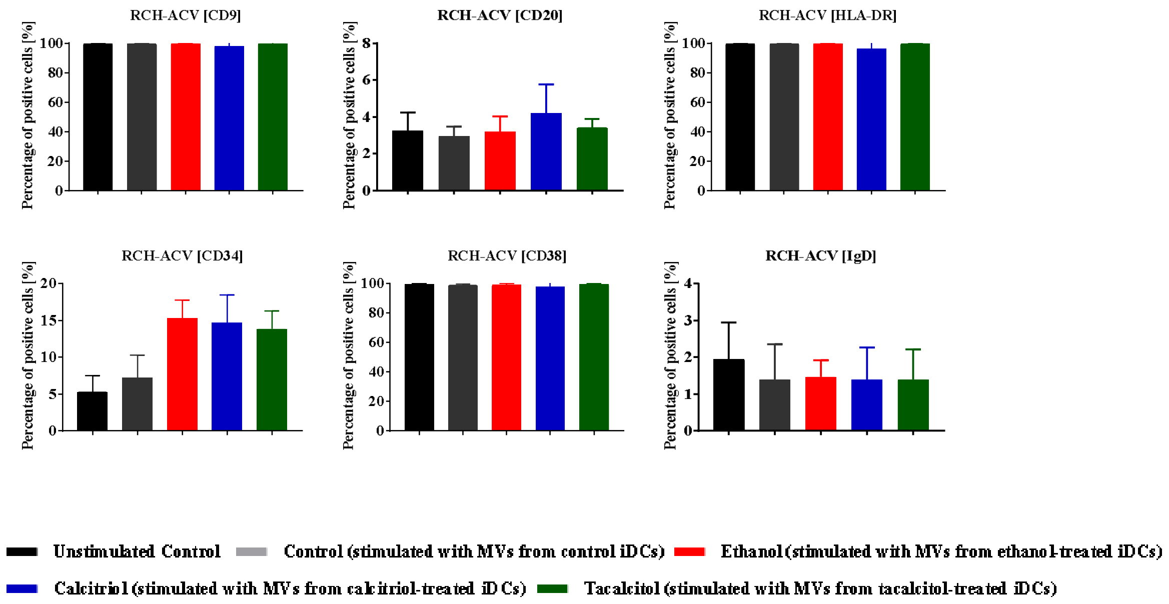

Exosomes

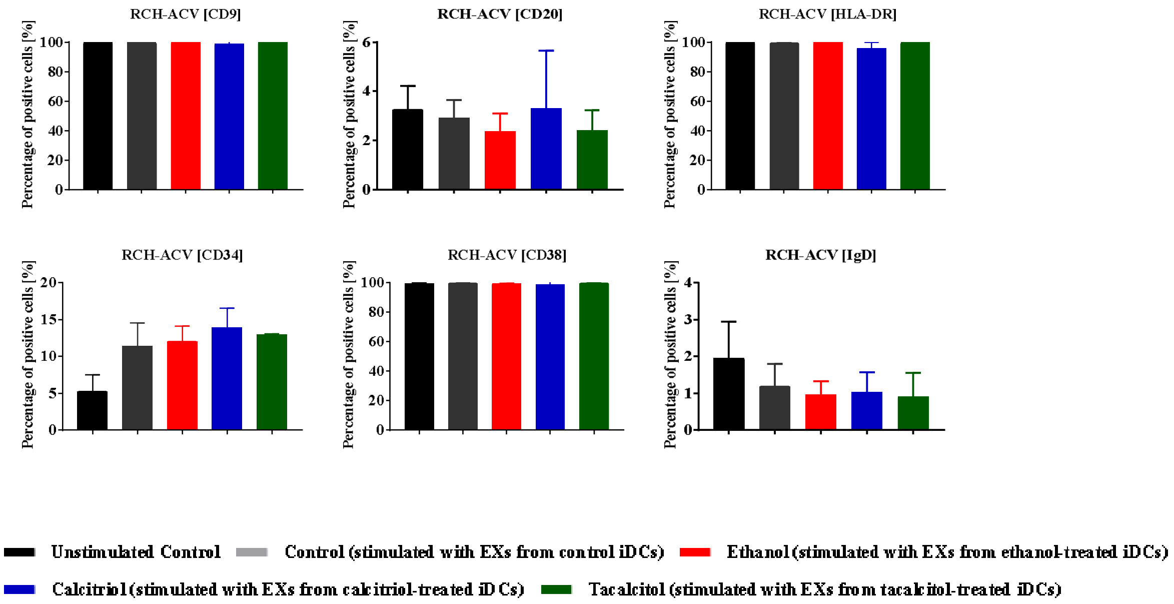

Figure S9. The indirect effect of calcitriol and tacalcitol on RCH-ACV cell line after 24 h of stimulation with myeloid dendritic cells - derived EVs (distribution of differentiation markers by flow cytometry analysis).

Mean values and standard deviation are presented. Statistical analysis: Sidak's multiple comparison in comparison to ethanol were performed. The graphs represent data from at least three separate experiments.

Microvesicles

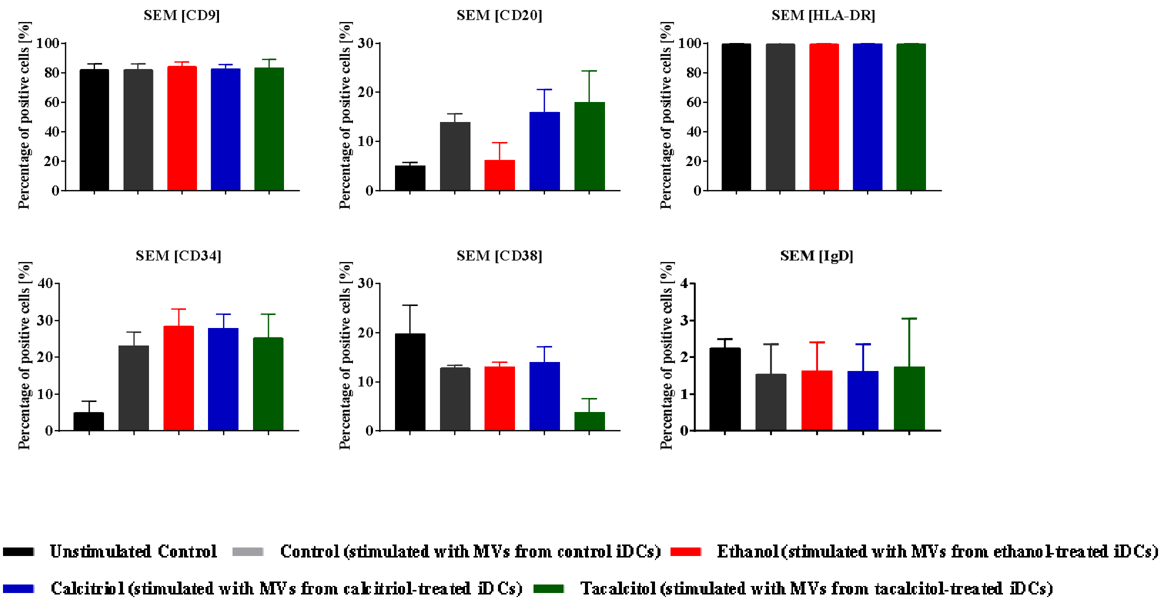

Exosomes

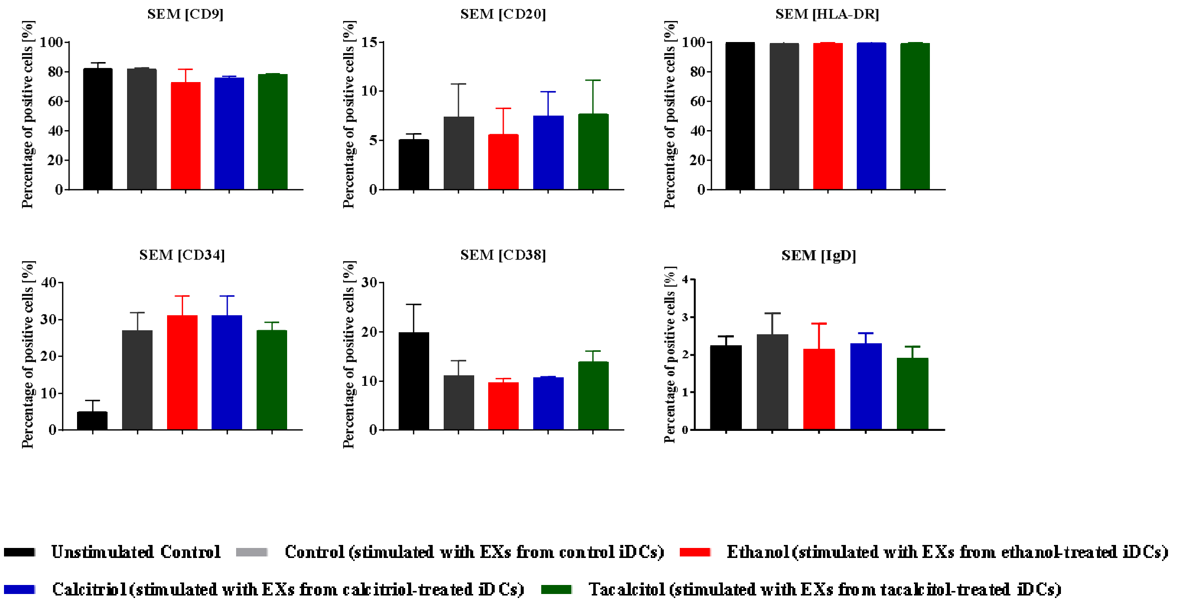

Figure S10. The indirect effect of calcitriol and tacalcitol on SEM cell line after 24 h of stimulation with myeloid dendritic cells - derived EVs (distribution of differentiation markers by flow cytometry analysis).

Mean values and standard deviation are presented.

Statistical analysis: Sidak's multiple comparison in comparison to ethanol were performed. The graphs represent data from at least three separate experiments.

Microvesicles

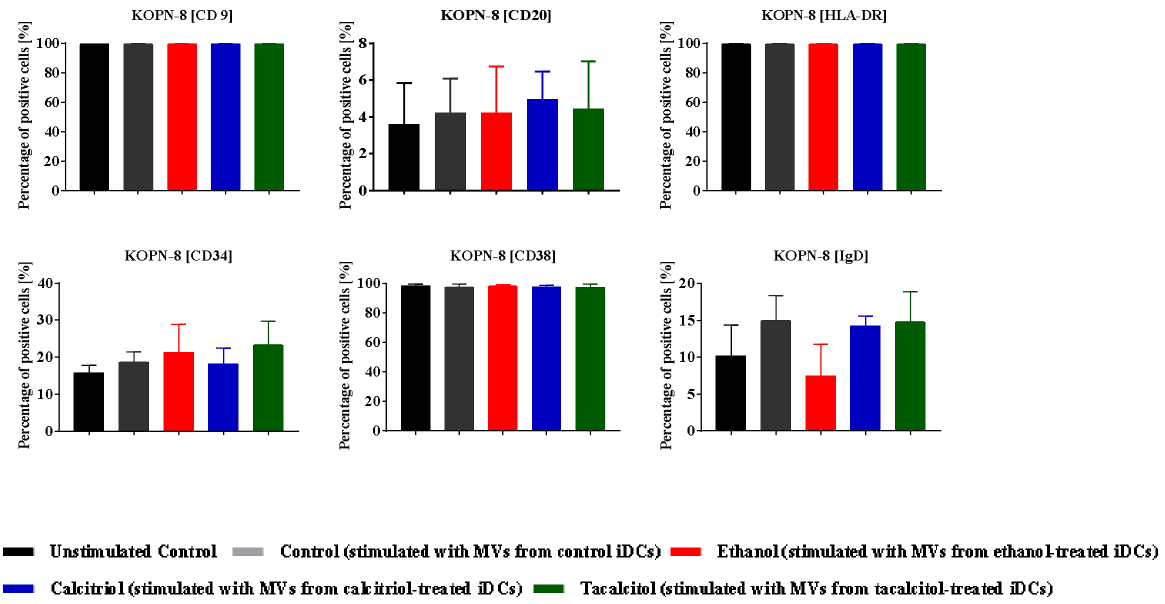

Exosomes

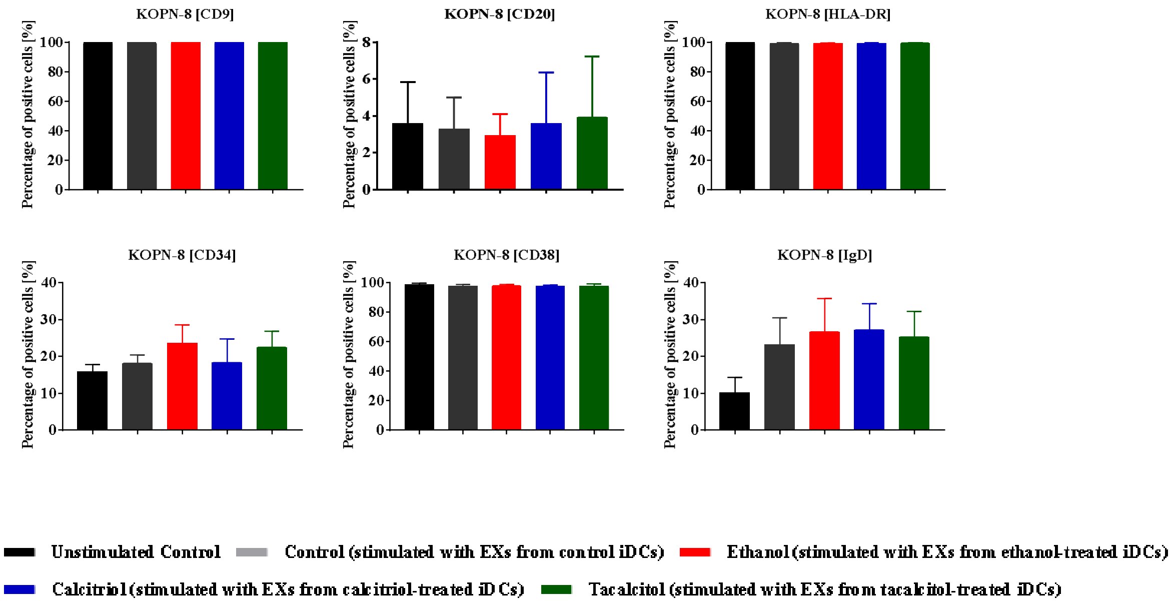

Figure S11. The indirect effect of calcitriol and tacalcitol on KOPN-8 cell line after 24 h of stimulation with myeloid dendritic cells - derived EVs (distribution of differentiation markers by flow cytometry analysis). Mean values and standard deviation are presented. Statistical analysis: Sidak's multiple comparison in comparison to ethanol were performed. The graphs represent data from at least three separate experiments.

Microvesicles

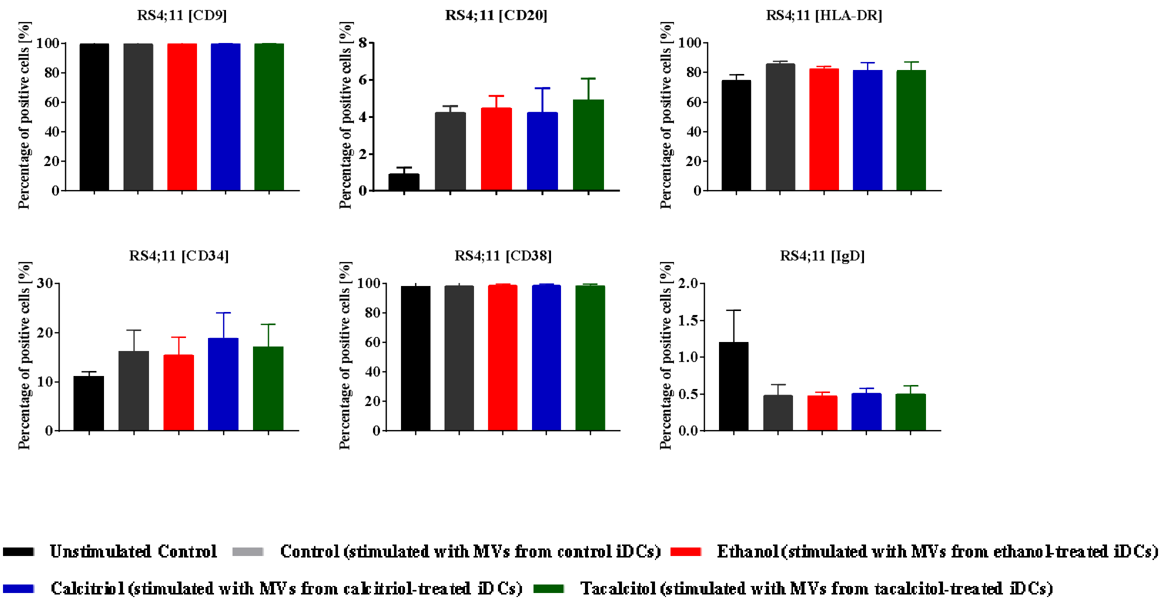

Exosomes

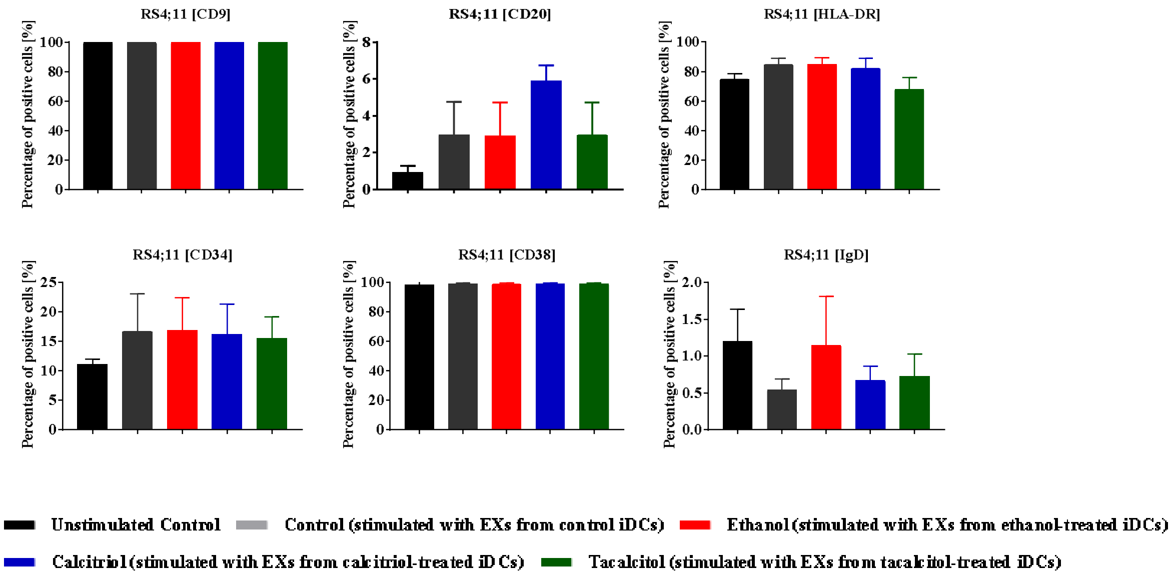

Figure S12. The indirect effect of calcitriol and tacalcitol on RS4;11 cell line after 24 h stimulation with myeloid dendritic cells - derived EVs (distribution of differentiation markers by flow cytometry analysis).

Mean values and standard deviation are presented. Statistical analysis: Sidak's multiple comparison in comparison to ethanol were performed. The graphs represent data from at least three separate experiments.

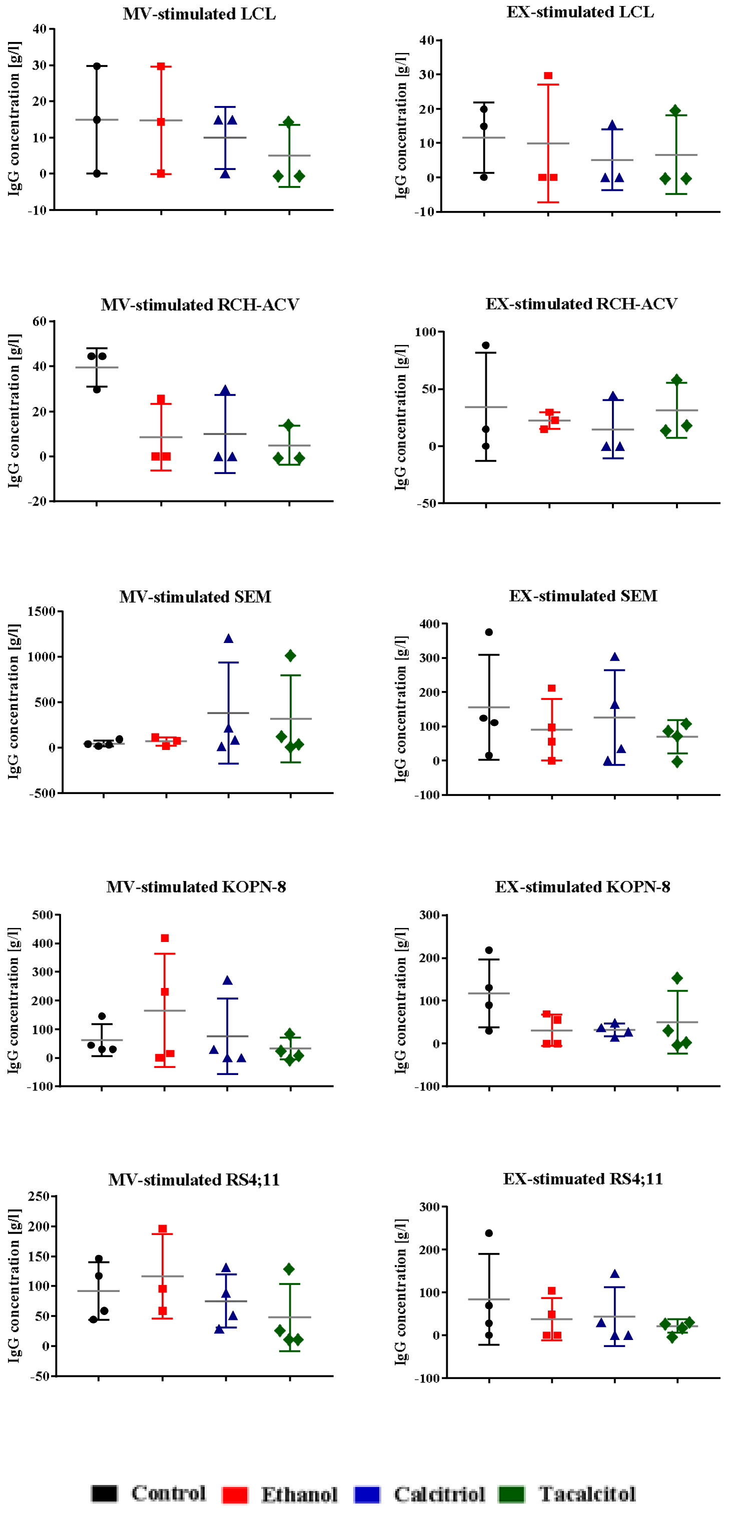

Figure S13. Total IgG concentration (g/l) after 24 h of stimulation with myeloid dendritic cells MVs/EXs.

Elisa measurement of total IgG concentration.

Mean values and standard deviation are presented. The graphs represent data from at least three separate experiments.

Table S1. The direct effect of calcitriol and tacalcitol on the distribution of CD markers on normal and leukemic B cells. Flow cytometry distribution of CD markers. Mean values and standard deviation are presented. The table represent data from at least three separate experiments.

**Cell Lines**	**Treatment**	**CD Markers Distribution [%]**						
		**CD9**	**CD10**	**CD20**	**CD23**	**CD34**	**IgD**	**HLA-DR**
**LCL**	**Control**	**90.55**±8.95	**28.23**±2.08	**91.36**±4.68	**98.65**±1.02	**80.31**±5.61	**49.36**±8.82	**99.05**±1.39
	**Ethanol**	**97.02**±1.74	**23.13**±5.78	**92.27**±4.42	**94.87**±5.51	**83.23**±5.73	**52.22**±4.79	**99.78**±0.20
	**Calcitriol**	**92.02**±4.70	**16.78**±5.49	**93.92**±1.88	**97.87**±1.13	**84.26**±3.18	**66.05**±6.73	**99.65**±0.26
	**Tacalcitol**	**97.85**±1.29	**17.42**±2.76	**95.10**±4.41	**95.92**±2.68	**91.69**±3.95	**50.89**±8.00	**99.71**±0.24
**RCH-ACV**	**Control**	**99.95**±0.04	**2.19**±1.98	**3.40**±0.54	**5.36**±1.08	**4.73**±1.23	**0.77**±0.10	**99.82**±0.05
	**Ethanol**	**99.96**±0.02	**1.31**±0.75	**3.37**±1.27	**5.62**±0.71	**9.13**±2.13	**0.75**±0.16	**99.72**±0.13
	**Calcitriol**	**99.95**±0.03	**2.59**±1.29	**5.02**±1.79	**6.98**±2.25	**5.33**±1.79	**0.71**±0.11	**99.71**±0.15
	**Tacalcitol**	**99.92**±0.04	**3.20**±1.35	**8.69**±1.63	**8.08**±0.71	**7.83**±2.68	**0.83**±0.25	**99.77**±0.09
**SEM**	**Control**	**77.51**±6.77	**2.57**±1.28	**9.66**±3.36	**3.17**±2.01	**6.90**±1.34	**2.91**±1.03	**99.55**±0.13
	**Ethanol**	**77.49**±8.64	**1.81**±0.66	**4.74**±0.98	**2.31**±1.46	**13.60**±5.74	**2.26**±0.28	**99.49**±0.18
	**Calcitriol**	**64.60**±8.34	**1.80**±0.65	**5.45**±1.99	**1.98**±0.89	**11.60**±2.41	**2.41**±0.58	**99.63**±0.26
	**Tacalcitol**	**65.73**±7.40	**1.49**±0.43	**5.66**±1.21	**1.46**±0.53	**10.67**±3.00	**2.46**±0.70	**99.75**±0.14
**KOPN-8**	**Control**	**99.93**±0.05	**2.43**±0.46	**9.16**±3.22	**8.25**±2.21	**19.15**±7.68	**3.87**±1.98	**98.93**±0.28
	**Ethanol**	**99.81**±0.09	**2.45**±0.17	**6.30**±0.90	**11.56**±0.95	**19.17**±8.48	**4.27**±1.16	**98.55**±1.10
	**Calcitriol**	**99.85**±0.10	**2.07**±0.85	**7.64**±1.55	**7.72**±1.78	**19.48**±6.14	**6.16**±1.27	**98.18**±0.71
	**Tacalcitol**	**99.82**±0.16	**2.29**±0.77	**7.54**±2.28	**9.11**±2.61	**16.39**±5.06	**2.08**±1.41	**99.28**±0.26
**RS4;11**	**Control**	**99.83**±0.15	**1.00**±0.36	**14.20**±4.82	**6.65**±2.43	**11.09**±0.92	**0.74**±0.17	**50.55**±6.30
	**Ethanol**	**99.80**±0.07	**3.06**±2.03	**13.32**±1.39	**4.43**±0.75	**11.13**±3.17	**1.03**±0.44	**50.89**±1.86
	**Calcitriol**	**99.88**±0.09	**2.35**±1.99	**14.39**±0.16	**6.67**±1.82	**11.45**±0.60	**0.83**±0.14	**44.36**±4.60
	**Tacalcitol**	**99.85**±0.05	**1.93**±0.69	**11.19**±1.94	**4.29**±1.27	**15.41**±4.26	**0.82**±0.14	**45.55**±3.06
